# Oversecretion and Overexpression of Nicotinamide Phosphoribosyltransferase/Pre-B Colony-Enhancing Factor/Visfatin in Inflammatory Bowel Disease Reflects the Disease Activity, Severity of Inflammatory Response and Hypoxia

**DOI:** 10.3390/ijms20010166

**Published:** 2019-01-04

**Authors:** Katarzyna Neubauer, Iwona Bednarz-Misa, Ewa Walecka-Zacharska, Jaroslaw Wierzbicki, Anil Agrawal, Andrzej Gamian, Malgorzata Krzystek-Korpacka

**Affiliations:** 1 Department of Gastroenterology and Hepatology, Wroclaw Medical University, 50-556 Wroclaw, Poland; kasianeu@gmail.com; 2 Department of Medical Biochemistry, Wroclaw Medical University, 50-368 Wroclaw, Poland; iwona.bednarz-misa@umed.wroc.pl (I.B.-M.); gamian@iitd.pan.wroc.pl (A.G.); 3 Department of Food Hygiene and Consumer Health, Wroclaw University of Environmental and Life Sciences, 50-375 Wroclaw, Poland; ewa.walecka@upwr.edu.pl; 4 Department of Minimally Invasive Surgery and Proctology, Wroclaw Medical University, 50-556 Wroclaw, Poland; jaroslaw.wierzbicki@umed.wroc.pl; 5 The 2nd Department of General and Oncological Surgery, Wroclaw Medical University, 50-556 Wroclaw, Poland; dranilpreeti@gmail.com

**Keywords:** Nicotinamide phosphoribosyltransferase (Nampt), pre-B factor (PBEF), visfatin, inflammatory bowel disease (IBD), Crohn’s disease, ulcerative colitis, mucosal healing, biomarker, hypoxia, epithelial-to-mesenchymal transition

## Abstract

Nicotinamide phosphoribosyltransferase’s (Nampt) association with inflammatory bowel disease (IBD) is unclear. The study was aimed at unraveling Nampt’s clinical and diagnostic relevance. The serum concentration (Luminex-xMAP® technology) was measured in 113 patients with Crohn’s disease (CD), 127 with ulcerative colitis (UC) and 60 non-IBD controls: 40 healthy individuals and 20 with irritable bowel syndrome (IBS). The leukocyte (44 CD/37 UC/19 IBS) and bowel expression (186 samples) was also evaluated (RT-qPCR). All were referred to IBD phenotype, activity, treatment, and inflammatory/nutritional/angiogenic/hypoxia indices. Serum-Nampt and leukocyte-*Nampt* were positively correlated and were more elevated in active-IBD than in IBS, with leukocyte-*Nampt* being a fair differential marker. Serum-Nampt in UC positively correlated with its clinical and endoscopic activity as well as with pro-inflammatory cytokines. Serum-Nampt ≤1.54 ng/mL was a good indicator of mucosal healing. The expression of *Nampt* was up-regulated both in inflamed and quiescent colon and reflected, similarly to leukocyte-*Nampt,* the clinical activity of IBD. Bowel-*Nampt* was independently associated with *IL1B* and hypoxia-inducible factor 1α *(HIF1A)* expression in inflamed bowel but with *FGF2* expression in quiescent bowel. In summary, Nampt’s elevation in IBD at local and systemic levels, and protein and mRNA levels, reflects IBD activity and is associated with inflammation, hypoxia (active) and tissue repair (inactive disease).

## 1. Introduction

Inflammatory bowel disease (IBD), which encompasses ulcerative colitis (UC) and Crohn`s disease (CD), refers to chronic, complex diseases of uncertain pathogenesis, currently affecting more than five million people worldwide [1,2,3]. IBD is characterized by a lack of a gold standard for diagnosis and monitoring [4]. The differential diagnosis is especially difficult as some non-inflammatory bowel conditions, e.g., irritable bowel syndrome (IBS), a model functional disorder of the digestive tract for which objective markers are non-existent, display a similar set of symptoms at the time of presentation [5,6].

Among several molecules, nicotinamide phosphoribosyltransferase (Nampt) has recently gained interest as a possible perpetrator of inflammation in IBD. To further address the issue, we aimed at expanding the current knowledge of clinical and diagnostic relevance of Nampt in IBD.

Nampt is a ubiquitously expressed [7] intracellular enzyme, which is involved in bioenergetics, cellular metabolism, adaptive stress responses, and DNA repair. Nampt catalyzes the formation of nicotinoamide mononucleotide (NMN) from nicotinamide (NAM) and phosphoribosyl -pyrophosphates, which is a rate-limiting step in the salvage pathway of nicotinamide dinucleotide (NAD) biosynthesis. Therefore, Nampt controls cellular NAD and NADH pools, and thus the redox status of the cell, and the activities of NAD-dependent enzymes. Despite lacking a classic secretory sequence, Nampt can be released into extracellular space, by mechanisms not fully resolved, where it acts as a growth factor and cytokine, named pre-B colony enhancing factor 1 (PBEF). In this form, Nampt displays hematopoietic, immunomodulatory, pro-inflammatory, pro-angiogenic, pro-chemotactic, and anti-apoptotic activities. In its extracellular form, it is also known as an adipokine visfatin, which demonstrates hormone-like insulin-mimicking properties [8,9].

It is becoming increasingly recognized that Nampt is a pleiotropic molecule playing an important role both in physiological and pathological processes. It is primarily associated with the induction and perseverance of inflammatory responses due to its ability to induce cytokine expression and secretion by various immune, epithelial and endothelial cells, to act as a chemokine, and to ensure survival of neutrophils, macrophages, and T lymphocytes [8,9,10]. Consequently, the elevation of circulating Nampt and/or its tissue expression accompanies a number of inflammatory-related conditions such as sepsis, rheumatoid arthritis, myocardial infarction, and cancer [8,9]. Nampt is gaining interest as a potential therapeutic target, especially in anti-cancer therapies [11,12]. Only recently has targeting Nampt been claimed as a ”promising therapeutic approach in acute intestinal inflammation” [13]. On the other hand, it is being considered a potential inflammation- and infection-associated biomarker [14,15,16].

Currently, available data on circulating Nampt in the adult IBD population are contradictory and mostly based on small cohorts [17,18,19,20]. The data on *Nampt*’s leukocyte [14] and bowel expression [17] are insufficient and lack detailed analysis. Therefore, our aim was a comprehensive analysis of Nampt in IBD patients at local and systemic levels, and at the protein and mRNA levels, which was conducted on a large cohort of patients and representative set of tissues. We intended to verify previous contradictory findings and evaluate, for the first time, the diagnostic utility of Nampt and its association with treatment. We also aimed at expanding the knowledge on leukocyte and bowel expression of *Nampt* beyond the notion of its up-regulation. This was done by evaluating the relationship between Nampt and disease activity and treatment, and by discerning the independent predictors of *Nampt* overexpression in the leukocytes and bowel tissue of IBD patients. We showed that Nampt is elevated in IBD at all examined levels and reflects IBD activity. Both serum- and leukocyte-*Nampt* show potential as biomarkers in IBD. Inflammation alone, or in combination with hypoxia, seems to be the main driving force of the over-secretion and over-expression of Nampt in active disease, whereas bowel over-expression of *Nampt* in quiescent tissue seems to be associated with tissue repair.

## 2. Results

### 2.1. Circulating Nampt

#### 2.1.1. Serum-Nampt (S-Nampt) in IBD

**Figure 1:**
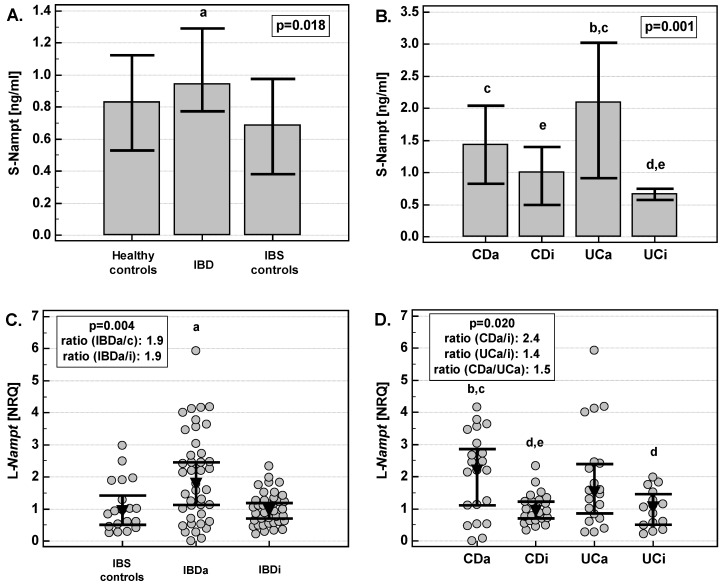
Nampt in IBD. (**A**) Comparison of S-Nampt in IBD, IBS- and healthy controls; (**B**) S-Nampt association with IBD phenotype and activity; (**C**) comparison of leukocyte expression of *Nampt* in active and non-active IBD and IBS-controls; (**D**) the association of leukocyte expression of *Nampt* with the disease phenotype and activity. Data are presented as medians with 95% confidence interval and analyzed using Kruskal–Wallis H test. Bars are used in panels A and B due to the presence of several cases with extremely high concentrations of S-Nampt, which, in the case of a dot-plot, make medians unreadable. S-Nampt, serum Nampt; IBD, inflammatory bowel disease; IBS, irritable bowel syndrome; CDa, active Crohn’s disease; CDi, inactive Crohn’s disease; UCa, active ulcerative colitis; UCi, inactive ulcerative colitis; L-*Nampt*, *Nampt* expression in leukocytes; NRQ, normalized relative quantities (fold-change against geometric mean across all investigated samples); IBDa/c, expression ratio of L-*Nampt* in active IBD to IBS-controls; IBDa/i, expression ratio of L-*Nampt* in active to inactive IBD; CDa/i, expression ratio of L-*Nampt* in active to inactive CD; UCa/i, expression ratio of L-*Nampt* in active to inactive UC; CDa/UCa, expression ratio of L-*Nampt* in active CD to active UC. a, significantly different from others; b, significantly different from CDi; c, significantly different from UCi; d, significantly different from CDa; e, significantly different from UCa.

S-Nampt in IBD patients was significantly higher than in IBS- and healthy controls (Figure 1A). Although the median concentrations between groups did not seem to differ by much, there was considerable patient-to-patient variation in the IBD group and 21% of IBD patients had S-Nampt exceeding maximal concentrations observed in both control groups (3.35 ng/mL). Over 15% of observations were classified as outside (*n* = 5) or far-out (*n* = 32) values.

There was no significant difference between inactive and active CD. However, patients with active UC had significantly more elevated S-Nampt than those with inactive disease (Figure 1B). Only patients with active CD or UC had significantly higher S-Nampt than healthy controls or IBS controls. In UC, S-Nampt positively correlated with the Rachmilewitz index (RI) (ρ = 0.28, *p* = 0.002) and the Mayo endoscopic score (ρ = 0.47, *p* < 0.0001). There were four UC patients with extremely high S-Nampt concentrations (>270 ng/mL). When excluded from analysis, the correlation coefficients were higher, respectively ρ = 0.36, *p* < 0.001 and ρ = 0.52, *p* < 0.0001 (see Supplementary data for graphs).

There was no significant difference in S-Nampt concentrations in patients with CD and UC, both when patients with inactive and active diseases were compared. However, patients with active UC tended to have higher S-Nampt concentrations than those with active CD (*p* = 0.194). When patients with active UC were compared with the combined patients with active CD and IBS, the difference in S-Nampt was significant (2.1 ng/mL (0.91–3.03) vs. 0.99 ng/mL (0.69–1.6), *p* = 0.029).

#### 2.1.2. S-Nampt Correlates with Nutritional and Inflammatory Markers

S-Nampt negatively correlated with iron, transferrin and albumin, and positively with indices of inflammation such as platelet count (in UC), leukocytes, erythrocyte sedimentation rate (in UC), and high sensitive C-reactive protein. There was no correlation with body mass index (Table 1). In UC, there was a tendency towards higher S-Nampt in patients with anemia (1.0 ng/mL (0.77–2.1) vs. 0.73 ng/mL (0.59–0.85), *p* = 0.054).

Correlation between S-Nampt and circulating interleukins (IL-1β, IL-6, IL-8), tumor necrosis factor α (TNFα), and vascular endothelial growth factor (VEGF)-A was examined in a subgroup of patients. In IBS-controls (*n* = 8), there was no correlation between cytokines. In IBD patients (*n* = 88), S-Nampt positively correlated exclusively with IL-6 (ρ = 0.30, *p* = 0.004). In IBD patients with active disease (*n* = 51), S-Nampt positively correlated with IL-6 (ρ = 0.52, *p* = 0.0001), IL-8 (ρ = 0.39, *p* = 0.005), and TNFα (ρ = 0.33, *p* = 0.018). Except for IL-6, which correlated with S-Nampt also in active CD (ρ = 0.44, *p* = 0.015; *n* = 30), the correlations were significant exclusively in UC (*n* = 21): r = 0.73, *p* < 0.001 (IL-6), r = 0.57, *p* = 0.008 (IL-8), r = 0.80, *p* < 0.0001 (TNFα) (see Supplementary data for graphs). Of these, TNFα was an independent predictor of S-Nampt concentration in multivariate analysis with r_partial_ = 0.80, *p* < 0.0001 and coefficient of determination (R^2^) = 0.64 (F = 33.5, *p* < 0.0001).

#### 2.1.3. S-Nampt Was More Elevated in UC Patients with Active Disease Treated with Azathioprine (AZA)

S-Nampt was significantly more elevated in patients with active CD and treated with corticosteroids (CS) than non-treated ones (2.26 ng/mL (1.24–4.42) vs. 0.80 (0.49–1.65), *p* = 0.010). However, when CS effect was co-examined with Crohn’s disease activity index (CDAI), it lost its significance (*p* = 0.115). The difference in S-Nampt between UC patients with active disease treated and non-treated with CS did not reach statistical significance (*p* = 0.115).

S-Nampt was significantly more elevated in UC patients with active disease treated than non-treated with AZA (3.43 ng/mL (2.1–15.1) vs. 0.8 (0.68–2.17), *p* = 0.001). When AZA effect was co-examined with RI, it remained significant (*p* = 0.033). There was no difference in S-Nampt related to AZA treatment in patients with inactive UC (*p* = 0.542).

#### 2.1.4. S-Nampt as a Biomarker in IBD

**Figure 2:**
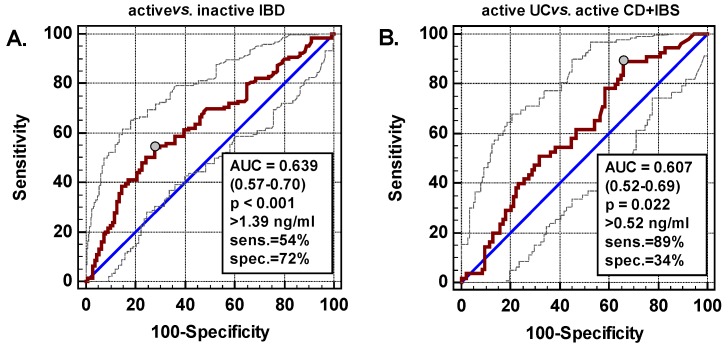

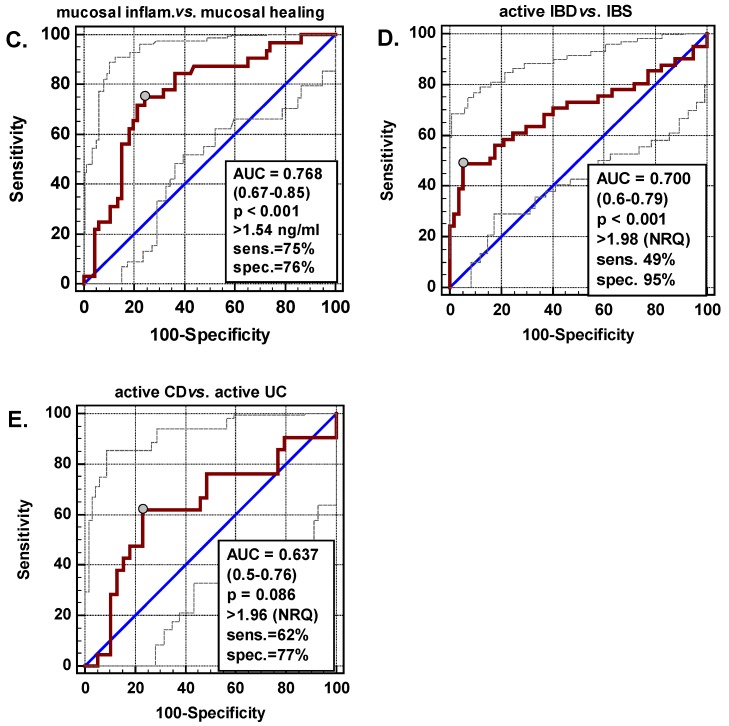
Nampt as an IBD marker. (**A**) S-Nampt as a marker differentiating patients with active and non-active disease; (**B**) S-Nampt as a marker differentiating patients with active UC from those with active CD and IBS; (**C**) S-Nampt as a marker differentiating UC patients with mucosal inflammation (Mayo endoscopic scores 2 and 3) from those without (scores 0 and 1); (**D**) L-*Nampt* as a marker differentiating IBD patients with active disease from IBS; (**E**) L-*Nampt* as a marker differentiating patients with active CD from active UC. Data are presented as ROC curves (straight line) with 95% confidence interval (CI) (dashed lines). Diagonal line presents the performance of a chance marker for which AUC = 0.5. Grey circle indicates optimal cut-off. Boxes contain data on the AUCs with 95%CI and probabilities of them being significantly different from a chance marker, optimal cut-offs with corresponding sensitivities and specificities. IBD, inflammatory bowel disease; CD, Crohn’s disease; UC, ulcerative colitis; IBS, irritable bowel syndrome; inflam., inflammation; AUC, area under receiver operating characteristic (ROC) curve; sens., sensitivity; spec., specificity.

Receiver operating characteristics (ROC) analysis was applied to evaluate S-Nampt as a potential marker in IBD. S-Nampt was significantly better than a chance marker (area under ROC curve (AUC) = 0.5) in discerning patients with active IBD from those with inactive disease (Figure 2A) and patients with active UC from those with active CD or IBS (Figure 2B). Yet, its overall accuracy was fair and S-Nampt displayed better specificity than sensitivity, except for differentiating active UC, in which case its sensitivity was high but was accompanied by poor specificity. However, S-Nampt was a good indicator of mucosal inflammation (endoscopic scores 2 and 3), displaying 77% overall accuracy and similarly high sensitivity (75%) and specificity (76%) (Figure 2C). Therefore, as a marker of mucosal healing (MH; endoscopic scores 0 and 1), S-Nampt ≤1.54 ng/mL was indicative of MH with 76% sensitivity and 75% specificity.

### 2.2. Leukocyte Expression of *Nampt*

#### 2.2.1. Leukocyte *Nampt* (L-*Nampt*) Expression Is Up-Regulated in Active IBD

**Figure 3:**
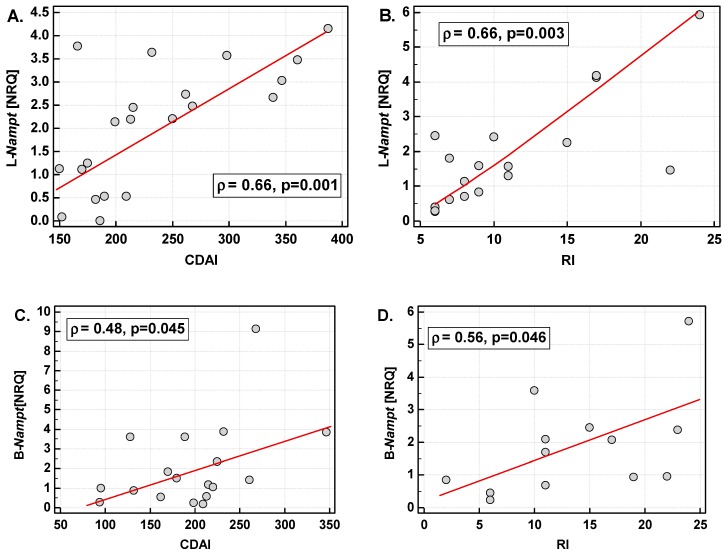
Correlation of *Nampt* with the disease activity. (**A**) L-*Nampt* with CDAI; (**B**) L-*Nampt* with RI; (**C**) B-*Nampt* in small intestine with CDAI; (**D**) B-*Nampt* in large intestine with RI. Data analyzed using Spearman rank correlation test. L-*Nampt*, Nampt expression in leukocytes; B-*Nampt*, *Nampt* expression in the intestine; NRQ, normalized relative quantities (fold change against geometric mean across all investigated samples); CDAI, Crohn’s disease activity index; RI, Rachmilewitz index.

Patients with active IBD had significantly higher L-*Nampt* than patients with inactive disease or controls (Figure 1C). In patients with active CD, L-*Nampt* expression was up-regulated by 2.4-fold on average, as compared to patients with inactive disease. In UC, the difference was 1.4-fold but did not reach statistical significance (*p* = 0.086) (Figure 1D). There was no difference in L-*Nampt* between controls and patients with inactive CD or UC. In active CD and UC, leukocyte expression was closely correlated with clinical activity of the diseases, expressed in terms of CDAI or RI (Figure 3A,B).

#### 2.2.2. L-*Nampt* Expression Correlates with Systemic Inflammatory and Nutritional Indices

L-*Nampt* negatively correlated with hemoglobin concentration and positively with platelets (in CD), leukocytes, and high sensitive C-reactive protein (in UC). It also positively correlated with leukocyte expression of *IL8*, exclusively in controls, and with *VEGFA* in CD and UC patients. No correlation with *IL1B* or *TNFA* expression was observed (Table 2).

Additionally, we examined the correlation between L-*Nampt* and circulating IL-1β, IL-6, IL-8, TNFα, and VEGF-A (data on cytokines were available for 39 patients, 25 with active IBD). In a whole group, L-*Nampt* positively correlated with all circulating cytokines: r = 0.52, *p* < 0.001 (IL-1β), r = 0.52, *p* < 0.001 (IL-6), r = 0.47, *p* = 0.003 (IL-8), r = 0.39, *p* = 0.013 (TNFα), and r = 0.33, *p* = 0.040 (VEGF-A) (see Supplementary data for graphs). In multiple regression, IL-1β (r_partial_ = 0.33, *p* = 0.043) and IL-6 (r_partial_ = 0.34, *p* = 0.037) were found independently associated with L-*Nampt* with R^2^ = 0.35 (F = 9.76, *p* < 0.001). In a subgroup of IBD patients with active disease, the correlation coefficients were even higher: r = 0.70, *p* = 0.0001 (IL-1β), r = 0.62, *p* < 0.001 (IL-6), r = 0.54, *p* = 0.006 (IL-8), r = 0.56, *p* = 0.004 (TNFα), and r = 0.42, *p* = 0.038 (VEGF-A) (see Supplementary data for graphs). In multiple regression, IL-1β (r_partial_ = 0.71, *p* = 0.0001) and VEGF-A (r_partial_ = 0.45, *p* = 0.026) were found independently associated with L-*Nampt* in IBD patients with active disease, with R^2^ = 0.59 (F = 15.96, *p* = 0.0001).

For 16 IBD patients, data on both S-Nampt and L-*Nampt* were available and there was a close correlation between both: r = 0.77, *p* < 0.001.

#### 2.2.3. L-*Nampt* Expression Is Up-Regulated in IBD Patients Treated with CS or AZA but the Effect Is Mediated by the Disease Activity

L-*Nampt* was significantly higher in patients treated with CS (2.14 ng/mL (1.26–2.58) vs. 1.1 ng/mL (0.83–1.24), *p* = 0.005) or AZA (1.54 ng/mL (1.11–2.37) vs. 1.11 ng/mL (0.77–1.29), *p* = 0.032). Significantly more patients with active than with inactive IBD were treated with CS (42 vs. 11%, *p* = 0.003) but not AZA (34 vs. 27%, *p* = 0.499). L-*Nampt* was significantly more up-regulated in active IBD patients treated than non-treated with CS (2.25 (1.59–3.46) vs. 1.12 (0.53–2.42), *p* = 0.028). When CD and UC patients were analyzed separately and CDAI or RI scores were included in the analysis, the effect of CS and AZA on L-*Nampt* lost its significance.

#### 2.2.4. L-*Nampt* as a Biomarker in IBD

Using ROC analysis, the suitability of L-*Nampt* as an IBD biomarker was evaluated. As a marker differentiating IBD patients with active disease from IBS, L-*Nampt* displayed a fair accuracy and poor sensitivity combined with excellent specificity. L-*Nampt*, as a marker differentiating patients with active CD from active UC, was not significantly better than a chance marker (Figure 2D,E).

### 2.3. Bowel Expression of *Nampt*

#### 2.3.1. Bowel *Nampt* (B-*Nampt*) Expression Is Up-Regulated Both in Inflamed and Quiescent Colon

**Figure 4:**
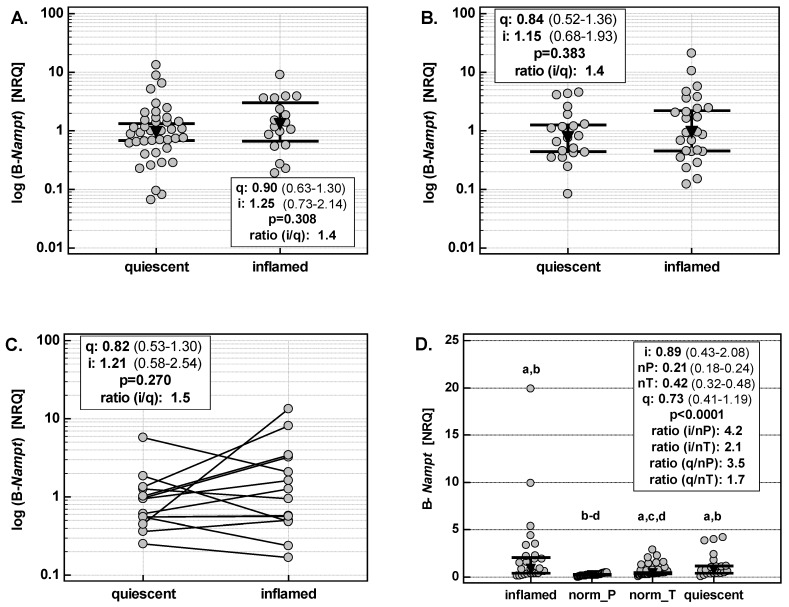
Nampt expression in the bowel. (**A**) B-*Nampt* in small bowel; (**B**) B-*Nampt* in large bowel; (**C**) B-*Nampt* in paired samples from the same patient; (**D**) B-*Nampt* in large bowel: comparison with normal tissues derived from patients with polyps or colorectal adenocarcinomas. B-*Nampt*, *Nampt* expression in the bowel; NRQ, normalized relative quantities (fold change against geometric mean across all investigated samples); norm_P, samples of macroscopically normal colonic tissue obtained from patients with polyps during endoscopy; norm_T, samples of macroscopically normal colonic tissue (resection margins) obtained from patients with colorectal adenocarinomas during curative resection of tumors; q, quiescent; i, inflamed; i/q, ratio of expression in inflamed to quiescent tissue; nP, normal tissue derived from patients with polyps; nT, normal tissue derived from patients with tumors; i/nP, ratio of expression in inflamed to normal tissue from patients with polyps; i/nT, ratio of expression in inflamed to normal tissue from patients with tumors; q/nP, ratio of expression in quiescent to normal tissue from patients with polyps; q/nT, ratio of expression in quiescent to normal tissue from patients with tumors. Data are presented as geometric means with 95% confidence interval (CI) and analyzed using *t*-test for unpaired samples (panels A and B) or *t*-test for paired samples (panel C) or as medians with 95%CI and analyzed with Kruskal–Wallis H test (panel D). a, significantly different from norm_P; b, significantly different from norm_T; c, significantly different from inflamed; d, significantly different from quiescent.

B-*Nampt* expression in inflamed small and large bowel was up-regulated by 1.4-fold as compared to quiescent mucosa of both small and large bowel (Figure 4A,B). A similar level of up-regulation was observed when B-*Nampt* expression was compared between inflamed and quiescent tissue from the same patient (Figure 4C). However, in both cases, the difference in B-*Nampt* did not reach statistical significance.

Additionally, we compared B-*Nampt* expression in the colons of IBD patients with its expression in macroscopically normal colonic tissue obtained from colorectal adenocarcinoma (CRC) patients or patients with polyps. B-*Nampt* expression in quiescent tissue was significantly up-regulated as compared to normal tissue from both patients with polyps (by 3.5-fold) and with colorectal cancer (by 1.7-fold). B-*Nampt* expression in inflamed tissue was up-regulated as well, respectively by 4.2- and 2.1-fold (Figure 4D).

B-*Nampt* expression in inflamed small and large bowels positively correlated with the clinical activity of CD and UC (Figure 3C,D).

#### 2.3.2. B-*Nampt* Expression Is Independently Associated with *IL1B* and *HIF1A* in Inflamed but with *FGF2* in Quiescent Bowel

In inflamed small bowel, B-*Nampt* positively correlated with the expression of *IL1B* (r = 0.85, *p* < 0.0001), *TNFA* (r = 0.84, *p* < 0.0001), *VEGFA* (r = 0.70, *p* = 0.001), *IL8* (r = 0.80, *p* = 0.0001), and *HIF1A* (r = 0.90, *p* < 0.0001) but not that of *FGF2* (r = 0.44, *p* = 0.070) (see Supplementary data for graphs). In multiple regression, *IL1B* (r_partial_ = 0.84, *p* < 0.0001) and *HIF1A* (r_partial_ = 0.89, *p* < 0.0001) were independent predictors of B-*Nampt* with R^2^ = 0.94 (F = 125.2, *p* < 0.0001).

In quiescent small bowel, B-*Nampt* positively correlated with the expression of *IL1B* (r = 0.76, *p* < 0.0001), *TNFA* (r = 0.57, *p* = 0.0001), *VEGFA* (r = 0.62, *p* < 0.0001), *IL8* (r = 0.72, *p* < 0.0001), *HIF1A* (r = 0.67, *p* < 0.0001), and *FGF2* (r = 0.62, *p* < 0.0001) (see Supplementary data for graphs). In multiple regression, *IL1B* (r_partial_ = 0.61, *p* = 0.006) and *FGF2* (r_partial_ = 0.66, *p* = 0.002) were independent predictors of B-*Nampt* with R^2^ = 0.76 (F = 26.12, *p* < 0.0001).

In inflamed large bowel, B-*Nampt* positively correlated with the expression of *IL1B* (r = 0.86, *p* < 0.0001), *TNFA* (r = 0.71, *p* = 0.0001), *VEGFA* (r = 0.56, *p* = 0.003), *IL8* (r = 0.84, *p* < 0.0001), and *HIF1A* (r = 0.70, *p* = 0.0001) but not *FGF2* (r = 0.13, *p* = 0.538) (see Supplementary data for graphs). In multiple regression, *IL1B* (r_partial_ = 0.87, *p* < 0.0001) and *HIF1A* (r_partial_ = 0.76, *p* < 0.0001) were independent predictors of B-*Nampt* expression with R^2^ = 0.89 (F = 87.93, *p* < 0.0001).

In quiescent large bowel, B-*Nampt* positively correlated with *IL1B* (r = 0.47, *p* = 0.038), *TNFA* (r = 0.77, *p* = 0.0001), *VEGFA* (r = 0.46, *p* = 0.040), *HIF1A* (r = 0.64, *p* = 0.003), and *FGF2* (r = 0.76, *p* = 0.0001) (see Supplementary data for graphs). In multiple regression, *TNFA* (r_partial_ = 0.58, *p* = 0.009) and *FGF2* (r_partial_ = 0.56, *p* = 0.012) were independent predictors of B-*Nampt* expression with R^2^ = 0.72 (F = 21.63, *p* < 0.0001).

## 3. Discussion

Nampt emerges as an important player in the pathogenesis of various diseases and its inhibition is considered an attractive therapeutic option. However, data on extracellular Nampt yielded by observational studies remain contradictory and the enzyme/cytokine suitability as a biomarker remains uncertain [9]. The strength of our study lies in a large number of enrolled patients as well as study comprehensiveness as Nampt was analyzed both at the local and systemic level, and both at the protein and mRNA level. We showed herein that active IBD is associated with S-Nampt elevation at all these levels. So far, available reports concerning circulating Nampt yielded inconsistent results, with both insufficient [18,19] and significant increases [17,20,21] being reported. Such discrepancy may result from considerable patient-to-patient variation. In our cohort of 240 IBD patients, median S-Nampt was around 1 ng/mL but over 15% of patients had outside/far-out values. The maximum concentration observed was almost four orders of magnitude higher than the median. With such variability, studies relying on small number of observations are likely to differ in their findings as they would probably be underpowered. The power /sample size estimation analysis conducted showed that for the comparison of means in healthy controls and diseased individuals with IBD, the study had to consist of over 180 patients due to high standard deviation. The negative study of Terzoudis et al. [19], in turn, suffered from the underrepresentation of patients with active disease. It had biased results as we and others [21] showed S-Nampt elevation to accompany the disease flare without significant difference between non-IBD individuals and IBD patients with inactive disease. Also, in other conditions, an exclusive association with Nampt in severe or acute inflammation has been reported [13,14,15,22,23].

Neither we, nor others [17,18,20,21], could confirm S-Nampt elevation in CD as compared to UC [19]. However, there were differences between both IBD phenotypes with respect to S-Nampt association with disease activity or treatment and correlation with inflammatory cytokines. Corroborating the observations of Moschen et al. [17], there was a significant difference in S-Nampt between patients with active and inactive disease exclusively in UC. Moreover, S-Nampt positively, although weakly, correlated with the clinical activity score (RI). A stronger relation between S-Nampt and the Mayo endoscopic score prompted us to evaluate it as a potential marker of mucosal healing (MH). MH is considered a key objective in the management of IBD patients. While currently applied methods for its evaluation, such as endoscopy, are sufficiently accurate, they are also either invasive or expensive and thus not optimal for regular monitoring of IBD patients [24]. Consequently, surrogate markers that would be non-invasive, easily measured and inexpensive, are intensively looked for [25]. In this capacity, S-Nampt displayed good overall accuracy and similarly good sensitivities and specificities. Since S-Nampt was significantly higher in patients with active UC than patients with active CD and IBS (combined), its value as a potential differential marker was evaluated as well. There is clinical demand for differential markers since both IBD phenotypes during the disease flare, as well as IBS, manifest themselves in a similar manner [6]. However, although better than a chance marker, S-Nampt as a differential UC marker displayed only fair overall accuracy, which excluded its practical application.

Mesko et al. [14] showed an up-regulated *Nampt* expression in leukocytes from patients with autoimmune diseases, IBD among others. Augmenting their research, we demonstrated L-*Nampt* to be elevated solely in active IBD. However, unlike S-Nampt, the up-regulation of L-*Nampt* was more evident in CD. Also, it is positively correlated with the clinical activity of both CD and UC. Supporting the notion on the significant contribution of leukocytes during inflammatory response to the increasing pool of circulating Nampt [15], S-Nampt and L-*Nampt* were strongly and positively correlated in our patients. There was also a positive correlation between S-Nampt and L-*Nampt* and leukocyte count as well as other indices of inflammation. Since whole blood for transcriptome analysis is easily and non-invasively obtained, the suitability of L-*Nampt* as a marker differentiating between active IBD and IBS as well as between active CD and UC was evaluated. Only as an active IBD marker, L-*Nampt* was better than a chance marker, displaying fair overall accuracy and near-perfect specificity but poor sensitivity.

Moschen et al. [17] reported an up-regulated expression of *Nampt* in inflamed as compared to normal but not quiescent colon in IBD patients. Corroborating their finding on a larger set of tissues that also included small bowel samples, we found B-*Nampt* to be only insignificantly up-regulated in inflamed as compared to quiescent bowel. The degree of the inflammation-associated up-regulation of B-*Nampt* was comparable between the small and large intestine. Moschen et al. [17] used biopsy samples from healthy controls undergoing screening colonoscopy as a reference. Here, we compared B-*Nampt* expression in the colons of IBD patients with tumor-free resection margins of CRC patients as well as biopsies of macroscopically normal tissue from patients with adenomas. B-*Nampt* expression in inflamed colon was significantly up-regulated by over 2-fold when referred to CRC-derived normal tissue and by over 4-fold when referred to adenoma-derived normal tissue. Interestingly, quiescent colonic tissue overexpressed B-*Nampt* as well, by 1.7-fold when referred to CRC-derived normal tissue and by 3.5-fold when referred to adenoma-derived normal tissue. Of note, we previously showed that *Nampt* expression in non-tumor tissue, although lower than in tumors, reflected CRC advancement just like tumor tissue [26] and thus, the non-involved tissue derived from patients with polyps is more likely to reflect physiological levels of colonic *Nampt* expression. The role of B-*Nampt* overexpression in quiescent colon may be a part of physiological tissue repair, or a pathological one leading to fibrosis, or simply precede the disease flare. The issue is further complicated by the fact that the analysis of *Nampt* expression does not provide an answer as to whether its overexpression would affect intracellular or extracellular pools of Nampt and their functionalities are not identical. Moschen et al. [17] identified various immune cells, apart from endothelial ones, as a source of Nampt in inflamed colonic tissue. Through immune cells, both extracellular and intracellular Nampt would exacerbate inflammation either by inducing the expression and secretion of inflammatory cytokines and chemokines and by preventing their apoptosis (extracellular Nampt) or by helping to sustain increased energetic demands of activated immune cells (intracellular Nampt) [9,27]. However, extracellular Nampt has also been shown to promote the epithelial-to-mesenchymal transition (EMT) [9], playing an important role in physiological tissue repair. Accordingly, B-*Nampt* expression exclusively in quiescent bowel (small and large) positively correlated with that of *FGF2*, a growth factor crucial for the repair of bowel injury [28]. Moreover, *FGF2* was independently associated with B-*Nampt* expression, explaining 76% in its variability in small bowel (together with *IL1B*) and 72% in large bowel (together with *TNFA*). Correlation analysis does not provide an answer to whether *Nampt* is among a number of repair genes activated by FGF2 to commence healing or whether it is Nampt up-regulating *FGF2* expression to promote angiogenesis [29]. Regardless of the direction of Nampt-FGF2 association, its effect seems to be regeneration.

However, pathologically sustained inflammation may lead to early and prolonged EMT and fibrosis [30]. Intestinal fibrosis is stimulated by TNF-α, which accelerates collagen production by intestinal fibroblasts and the secretion of matrix metalloproteinases but reduces myofibroblast mobility [31]. In this respect, it is of interest that in quiescent colonic tissue *TNFA* expression was independently associated with that of *Nampt*. Fibrosis in CD has been associated with enhanced proliferation of smooth muscle cells [32]. While there are no studies on Nampt association with intestinal fibrosis, Pillai et al. [33] demonstrated that sustained activation of Nampt causes cardiomyocyte hypertrophy and increased the growth and differentiation of cardiac fibroblasts to myofibroblasts. These authors concluded that the intentional effect of Nampt is cell protection against oxidative stress and hypoxia but its chronic elevation leads to adverse consequences. It stands to reason that, in case of bowel and Nampt, the undesirable consequences of its sustained overexpression might be intestinal fibrosis. Moreover, it may also increase the risk of neoplastic transformation, one of the most serious complications of IBD. In addition to sustaining proinflammatory milieu and being involved in EMT, Nampt promotes the protumorigenic phenotype in resident macrophages [9]. Cancer-associated EMT is triggered by inflammation and hypoxia [34], and we showed the overexpression of *Nampt* in the inflamed intestine to be associated with both. Moreover, *IL1B* and *HIF1A* were independent predictors of B-*Nampt* expression, explaining 94% and 89% of its variability in, respectively, small and large bowel. Hypoxia has been demonstrated to induce *Nampt* expression in various cell types (reviewed in [27]) and two functional HIF-1α responsive elements (HRE) have been found in the promoter region of a gene encoding Nampt [35]. Also, co-expression of *Nampt* and *HIF1A* has previously been shown in colon and peripheral leukocytes of CRC patients [26]. Our finding implies that hypoxia, through HIF-1α, might be a driving force of *Nampt* expression in inflamed bowel of IBD patients. Supporting the notion of Nampt contribution to colitis-associated cancer, inhibition of *Nampt* in intestinal mucosa in mice models of colitis has reduced local inflammatory response and blocked inflammation-associated neoplastic transformation [13].

In line with a close and bidirectional relation between Nampt and inflammation [9,13,17,22], Nampt in our study correlated with prototypical inflammatory cytokines, IL-1b, IL-6, and TNFα as well as with IL-8 and VEGF-A. In circulation, the association was the strongest with IL-6 as it was present across the whole IBD cohort. This finding is consistent with the fact that the *Nampt* promoter region contains IL-6 response elements in addition to classic regulatory elements crucial for cytokine up-regulation, such as NFκB, AP-1, and NF-1 [22]. The correlations with other cytokines were significant exclusively in patients with active UC, for whom TNFα was found to be an independent predictor of circulating Nampt, explaining 64% in its variability. In turn, the up-regulation of leukocyte expression of *Nampt* was independently associated with IL-1b and IL-6, the latter cytokine being replaced by VEGF-A in patients with active disease.

Corticosteroids are common medication in IBD treatment used to reduce the inflammatory response. They were also found to reduce *Nampt* up-regulation induced by pro-inflammatory cytokines [22]. As the *Nampt* promoter contains several binding sites for the glucocorticoid receptor, it was speculated that *Nampt* expression may be directly down-regulated by corticosteroids [22]. However, subsequent in vitro studies have shown that steroids up-regulate *Nampt* in adipocytes (dexamethasone) [36] and fibroblasts (estrogens) [37] and that their effect is synergistic [37]. In animal studies, *Nampt* expression has been demonstrated to be up-regulated by estrogen but down-regulated by progesterone [38]. As in the case of cytokines, Nampt interaction with steroids seems to be bidirectional: in rat models, peritoneal administration of Nampt increased serum concentrations of corticosterone and Nampt has been implicated in the regulation of the hypothalamic-pituitary-adrenal axis [39]. In clinics, there are only a few reports on the relationship between steroid therapy and Nampt, and their results remain inconclusive. Inhaled fluticasone has no effect on Nampt in patients with chronic obstructive pulmonary disease [40] but, in combination with mycophenolate mofetil, it has induced a discreet reduction in circulating Nampt in patients with systemic lupus erythematous (SLE) [23]. On the other hand, there has been a positive correlation between the duration of steroid therapy and Nampt concentration in SLE patients [23]. In CD, but not UC, treatment (CS, AZA) induced a decrease in the serum Nampt concentrations [20]. We, in turn, observed an up-regulated Nampt on systemic and leukocyte levels in patients treated with CS, particularly with CD, and with AZA, particularly with UC. However, except for the AZA effect on circulating Nampt in patients with active UC, treatment effect seems to be mediated by the severity of the disease. Possible association between CS treatment and Nampt elevation is of interest as it may provide a link between CS-induced osteoporosis [41] in IBD patients as an elevation in Nampt has been shown to be associated with this extraintestinal manifestation of IBD [19,42].

As sample collections for our study were conducted in different time periods and/or different clinics (except for 16 patients assessed for both serum concentration and leukocyte expression of Nampt), it was not possible to analyze the relation between circulating, leukocyte and bowel Nampt, which is a limitation of our study. Attention might also be drawn to the large disproportion of the initial size of groups in the serum-Nampt part of the study (240 IBD patients, 40 healthy controls and 20 IBS patients). While an initially large IBD cohort is understandable as it allows for analysis in subgroups to retain reasonable power, the relatively low number of non-IBD controls might be considered another limitation. However, such a disproportionate study design was in fact dictated by the power analysis/sample size estimation. Contrary to the IBD group, characterized by extreme inter-individual variation, standard deviation in S-Nampt in both non-IBD control groups is low and does not necessitate (and justify) the enrollment of more individuals than recruited.

## 4. Materials and Methods

### 4.1. Study Population

The study consists of three parts (serum, leukocyte and bowel Nampt), conducted in different time periods and in different departments. Patients were separately enrolled for each part and are described in the following paragraphs in a separate manner as well. Only 16 patients were examined for both serum and leukocyte Nampt.

#### 4.1.1. Circulating Nampt

S-Nampt was evaluated in 300 individuals: 240 IBD patients, 40 healthy controls and 20 IBS-controls. IBD and IBS patients were admitted to the Department of Gastroenterology and Hepatology of Wroclaw Medical University due to the disease diagnosis, flare-up or monitoring (IBD) or for diagnosis (IBS). Apparently healthy controls (unremarkable medical history, no known systemic diseases, no current infections, no pregnancy, and no treatment of any type) were volunteers recruited from the hospital staff. From among IBD patients, there were 113 individuals with CD (74 with active disease) and 127 with UC (55 with active disease). Inclusion criteria consisted of age ≥18 years, diagnosed or suspected IBD (patients in whom IBS instead of IBD was diagnosed formed the IBS group) and willingness to participate. Patients with indeterminate colitis, celiac disease or other functional and inflammatory bowel disorders or with the co-existence of other severe systemic diseases, malignancies, liver diseases, renal failure, diabetes or pregnancies were not included. For the assessment of CD and UC activity, respectively, the Crohn’s Disease Activity Index (CDAI) and the Rachmilewitz Index (RI) were used. Endoscopic findings in UC were evaluated according to the Mayo Scoring System for Assessment of Ulcerative Colitis Activity [43]. Almost all IBD patients were treated with 5′-aminosalicylate (5′-ASA) derivatives. Additionally, corticosteroids (CS) were administered in 93 patients, and azathioprine (AZA) in 77 IBD patients. Anemia was defined according to the WHO criteria as hemoglobin concentrations below 13 g/dL in males and 12 g/dL in females. The study population did not differ significantly with respect to age and sex distribution. IBD patients had lower concentrations of hemoglobin, iron, transferrin, and albumin and higher erythrocyte sedimentation rate (ESR), high-sensitive C-reactive protein, and IL-6 (for detailed characteristics see Table 3).

#### 4.1.2. Leukocyte *Nampt* (L-*Nampt*)

Leukocyte expression of *Nampt* was evaluated in 98 individuals: 79 IBD patients and 19 IBS patients who served as controls. All individuals were recruited from the Department of Gastroenterology and Hepatology of Wroclaw Medical University. There were 44 patients with CD (21 with active disease) and 35 with UC (20 with active disease). The study population did not differ significantly with respect to age and sex distribution. IBD patients had lower hemoglobin and higher platelet counts and ESRs than the IBS-control group (for detailed characteristics see Table 4).

#### 4.1.3. Bowel *Nampt* (B*-Nampt*)

Bowel expression of *Nampt* was evaluated in 186 tissue samples. Of these, 104 samples were collected from 52 IBD patients with 58 tissue samples harvested from small bowel and 46 tissue samples from large bowel. Small bowel samples were obtained from inflamed (*n* = 18; CD patients) or quiescent bowel (*n* = 40: 18 from CD and 22 from UC patients). Large bowel samples were obtained from inflamed (*n* = 26: 10 from CD and 16 from UC patients) or quiescent bowel (*n* = 20: 18 from CD and 2 from UC patients). Paired tissue samples were available for 14 CD patients: 10 with ileal CD and four with colonic CD. UC patients had pancolitis and there was no quiescent colonic tissue to be obtained for paired (patient-matched) analysis. IBD patients were admitted to the First Dept. and Clinic of General, Gastroenterological and Endocrinological Surgery of Wroclaw Medical University due to the ineffectiveness of pharmacological therapy or disease complications, such as perforation, obstruction, fistula, abscess, or in order to complete further stages of previous surgery procedure. Additionally, for comparative purposes, 48 tissue samples from tumor-free (histologically confirmed) resection margins from patients with colorectal adenocarcinoma (CRC) undergoing curative tumor resection in the Dept. of Gastrointestinal and General Surgery of Wroclaw Medical University as well as normal tissue samples from 34 patients undergoing polypectomy (histologically confirmed adenomas) in the Dept. of Minimally Invasive Surgery and Proctology were used as reference.

#### 4.1.4. Ethical Considerations

The study protocol was approved by the Medical Ethics Committees of Wroclaw Medical University (KB-71/2007 from 15 February 2007 for the part on circulating Nampt and KB-575/2011 from 10 November 2011 for expression studies) and the study was conducted in accordance with the Helsinki Declaration of 1975, as revised in 1983, and informed consent was obtained from all patients.

### 4.2. Analytical Methods

#### 4.2.1. Circulating Nampt

Blood samples were drawn by venipuncture into BD Vacutainer CAT tubes (Becton Dickinson, Plymouth, UK) following overnight fasting and centrifuged (15 min., 720× *g*). Serum samples were collected, aliquoted, and stored at −80 °C until examination. S-Nampt was measured in duplicates by means of the flow cytometry-based method utilizing magnetic microspheres conjugated with monoclonal antibodies using the BioPlex 200 (Bio-Rad, Hercules, CA, USA) platform, incorporating Luminex xMAP^®^ technology, and the following reagents purchased from Bio-Rad (Hercules, CA, USA): Bio-Plex ProTM Hu Diabetes Visfatin Set (#171B7012M), reagent kit (#171-304070), and visfatin standard (#171-D70001). The same technique and Bio-Plex Pro™ Human Cytokine 27-plex Assay (#M500KCAF0Y) were used to evaluate serum concentrations of IL-1β, IL-6, IL-8, TNFα, and VEGF-A in a subgroup of patients (39 in leukocyte *Nampt* study and 96 in circulating Nampt study). Standard curves were drawn using 5-PL logistic regression and the data were analyzed using BioPlex Manager 6.0 software.

#### 4.2.2. *Nampt* Expression in Leukocytes

Whole blood (3 mL) was collected into PAXgene Blood RNA Tubes and stored at −80 °C until RNA isolation. RNA was isolated using the complementary PAXgene Blood RNA Kit (Qiagen, Hilden, Germany) as instructed by the manufacturer. The concentration of isolated RNA was quantified using NanoDrop 2000 (ThermoScientific, Batavia, IL, USA) with the concomitant evaluation of RNA purity (ratios of absorbance at 260, 280, and 230 nm). RNA integrity was evaluated using the Experion platform incorporating LabChip microfluidic technology and Experion RNA StdSens analysis kits (BioRad, Hercules, CA, USA) and expressed as an RNA quality indicator (RQI) score with RQI = 1 indicative of degraded and RQI = 10 indicative of intact RNA. Only RNA isolates with RQI ≥ 7 were used for RT-qPCR. The possible presence of inhibitors in each RNA isolate was tested by calculating RT-qPCR efficiencies from standard curves prepared by serial dilutions of respective cDNA samples (five-fold dilutions, 6 point-curve, conducted in duplicates using SG qPCR Master Mix from EURx, Gdansk, Poland). A working dilution of cDNA 1:5 was found to effectively dilute reaction inhibitors and assure near 100% qPCR efficiencies.

#### 4.2.3. *Nampt* Expression in Bowel Tissue

Surgical tissue specimens were rinsed and soaked in RNAlater (Ambion Inc., Austin, TX, USA) and stored at −80 °C until RNA isolation. Tissue samples (30–40 mg) were homogenized using Fastprep 24 Homogenizer (MP Biomedical, Solon, OH, USA) in a lysis buffer (RLT buffer with a guanidine salt, part of RNeasy Plus Mini Kit from Qiagen, Hilden, Germany) with β-mercaptoethanol (Sigma-Aldrich, St. Louis, MO, USA). RNA was isolated from the homogenate using phenol–chloroform extraction and purified using RNeasy Plus Mini Kit (Qiagen, Hilden, Germany). Genomic DNA was removed by passing the sample through gDNA Eliminator spin columns and by additional on-column treatment of RNA isolates with DNase (Qiagen, Hilden, Germany). The concentration and quality of isolated RNA was assessed as described for leukocyte RNA.

#### 4.2.4. Reverse Transcription (RT)

An amount of 0.25 µg of RNA (whole blood samples) or 0.5 µg of RNA (tissue samples) per reaction (20 µL) was reversely transcribed in C1000 termocycler (BioRad, Hercules, CA, USA) using Maxima First Strand cDNA Synthesis Kit for RT-qPCR (Thermo Scientific, Batavia, IL, USA) and the protocol suggested by manufacturer. All samples were accompanied by matching negative transcription (“no-RT”) controls, devoid of reverse transcriptase, subsequently tested to assure the lack of contamination with genomic DNA.

#### 4.2.5. Quantitative (Real-Time) PCR (qPCR)

qPCRs were conducted using the CFX96 Real-Time PCR system (BioRad, Hercules, CA, USA) and SsoFast EvaGreen^®^ Supermix (BioRad, Hercules, CA, USA). The following cycling conditions were applied: 30 s activation at 95 °C, 5 s denaturation at 95 °C, annealing/extension for 5 s at 61 °C, 40 cycles, followed by melting step (60–95 °C with fluorescent reading every 0.5 °C). The reaction mixture contained 2 µL of cDNA (diluted 1:5), 10 µL of 2× SsoFast EvaGreen^®^ Supermix, 1 µL of each 10 nM forward and reverse target-specific primers, and water up to 20 µL. Only primers spanning at least one intron were used. Their specificities were tested by melting curve analysis and electrophoresis in a high-resolution agarose (SeaKem LE agarose, Lonza, Basel, Switzerland) in TBE with SYBR Green (Lonza, Basel, Switzerland) detection. Only primers yielding a single peak in melt curve and a single band in gel electrophoresis were used. Primers were also tested on no-RT samples with known contamination with gDNA and only these not amplifying gDNA at all or with no-RT signal at least 10 cycles apart from sample signal were selected. Primers’ efficiencies were assessed using a mixture of all cDNA samples as a template and calculated from a four-fold dilution series (six measuring points in triplicates), plotted as Cq vs. logarithms of dilution values of the DNA templates. The primers’ sequences and efficiencies are presented in Table 5. Primers were synthesized by Generi Biotech (Hradec Králové, Czech Republic). Samples were assessed in three technical replicates and accompanied by a “no template” control.

#### 4.2.6. Normalization Strategy, Calculation of Expression Level

Technical replicates were averaged and the efficiency-corrected Cq values were calculated for all examined genes and used in all subsequent calculations. For each sample, a geometric mean of Cq values obtained for two reference genes was calculated and subtracted from the Cq of the gene of interest (ΔCq). For paired sample analysis, the ΔCq values obtained for reference tissue were subtracted from the values obtained for matched inflamed tissue (ΔΔCq) and the relative level of expression (fold change in expression) was calculated as 2^(−ΔΔCq). For unpaired analysis, geometric means of all ΔCq values in a given analysis were obtained and a ratio of sample ΔCq to a group mean was calculated using qbasePLUS version 2.4 software (Biogazelle BE, Ghent, Belgium) and referred to as a normalized relative quantity (NRQ) [44].

The reference gene selection was based on our earlier results, showing *PPIA* and *RPS23* the most suitable pair of genes for studies on bowel tissues from IBD patients and *PPIA* and *RPLP0* for studies on bowel tissues from patients with colorectal adenocarcinomas [45]. For leukocyte analysis, *TBP* and *SDHA* were used as the genes that expressed relatively stable levels in leukocytes of IBD patients (manuscript submitted).

### 4.3. Statistical Analysis

Power analysis/sample size estimation was conducted to estimate the minimal number of individuals to be enrolled into the IBD, IBS, and healthy control groups for their mean comparison test to have acceptably low type I and II errors (α and β = 0.05).

The normality of distribution and homogeneity of variances was tested using Kolmogorov–Smirnov and Levene’s tests, respectively. Log-transformation was applied if suitable. Unpaired observations were analyzed using either t-test for independent samples and one-way ANOVA with the Tukey–Kramer post-hoc test (normally distributed data) or the Mann–Whitney U test and the Kruskal–Wallis H test with the Conover post-hoc test (non-normally distributed data). Outlying observations were detected using the Tukey method and classified as outside values (values that are smaller/larger than the lower/upper quartile minus/plus 1.5 times the interquartile range) and far-out values (values that are smaller/larger than the lower/upper quartile minus/plus 3 times the interquartile range). Bonferroni correction for multiple testing was applied. Paired observations were analyzed using *t*-test for paired samples. Data are presented as means or medians and accompanied by 95% confidence intervals (CI). Frequency analysis was conducted using the Fisher exact test (2 × 2) or Chi-square test. Correlation analysis was conducted using Pearson (r) or Spearman rank (ρ) correlation tests, depending on data character and distribution. Multiple regression (stepwise method) was used to discern variables independently associated with the dependent variable (Nampt concentration or expression). Analysis of co-variance was used to co-examine the effect of treatment and the disease severity. Receiver operating characteristic (ROC) curve analysis was conducted to assess the diagnostic power of circulating Nampt as an IBD marker. Overall marker accuracy, defined as area under ROC curve (AUC) expressed in %, was calculated and interpreted, based on likelihood ratios, as follows: AUC = 0.50 to 0.75 = fair accuracy; 0.75 to 0.92 = good accuracy; 0.92 to 0.97 = very good accuracy; and 0.97 to 1.00 = excellent accuracy. Additionally, optimal cut-off was established, and corresponding sensitivities and specificities were calculated. All tests were two-sided and *p* values <0.05 were considered statistically significant. Statistical analysis was conducted using MedCalc Statistical Software version 18.9 (MedCalc Software bvba, Ostend, Belgium; http://www.medcalc.org; 2018).

## 5. Conclusions

In this study, we showed that Nampt is elevated in IBD at local and systemic levels, protein and mRNA levels, and reflects IBD activity. Serum-Nampt or leukocyte-*Nampt* show potential for use as mucosal healing markers or markers differentiating between inflammatory vs. non-inflammatory bowel diseases, respectively. Inflammation or inflammation together with hypoxia seem to be the main driving forces of the over-secretion and over-expression of Nampt associated with active disease, whereas bowel over-expression of *Nampt* in quiescent tissue seems to be associated with tissue repair.

## Figures and Tables

**Table 1:** Correlation pattern of circulating Nampt/pre-B colony enhancing factor 1 (PBEF)/visfatin (S-Nampt).

Parameter	Non-IBD Controls		IBD	
	Healthy Individuals	IBS	CD	UC
BMI (kg/m^2^)	−0.44, *p* = 0.200	−0.34, *p* = 0.276	−0.08, *p* = 0.559	0.0, *p* = 0.940
Hb (g/L)	na	0.38, *p* = 0.226	0.01, *p* = 0.903	−0.17, *p* = 0.068
Iron (μM)	−0.29, *p* = 0.086	0.13, *p* = 0.681	−0.34, *p* = 0.003	−0.30, *p* = 0.006
Tf (mg/dL)	−0.02, *p* = 0.915	−0.02, *p* = 0.957	−0.28, *p* = 0.018	−0.34, *p* = 0.001
Alb (g/dL)	−0.20, *p* = 0.212	−0.14, *p* = 0.636	−0.35, *p* = 0.001	−0.28, *p* = 0.006
PLT (×10^9^/L)	na	−0.54, *p* = 0.089	0.14, *p* = 0.175	0.20, *p* = 0.030
WBC (×10^9^/L)	na	−0.39, *p* = 0.217	0.27, *p* = 0.009	0.18, *p* = 0.044
ESR (mm/h)	na	−0.20, *p* = 0.526	0.13, *p* = 0.219	0.41, *p* < 0.001
hsCRP (mg/L)	−0.10, *p* = 0.521	0.12, *p* = 0.744	0.35, *p* = 0.013	0.37, *p* = 0.002

Data are presented as Spearman correlation coefficients (ρ). BMI, body mass index; Hb, hemoglobin; Tf, transferrin; Alb, albumin; PLT, platelet count; WBC, white blood cell count; ESR, erythrocyte sedimentation rate; hsCRP, high-sensitive C-reactive protein; IBS, irritable bowel syndrome; IBD, inflammatory bowel disease; CD, Crohn’s disease; UC, ulcerative colitis; na, non-available.

**Table 2:** Correlation pattern of leukocyte Nampt/PBEF/visfatin (L-*Nampt*).

Parameter	Non-IBD Controls (IBS)	IBD	
		CD	UC
Hb (g/L)	ρ= −0.46, *p* = 0.047	ρ= −0.34, *p* = 0.025	ρ= −0.36, *p* = 0.032
PLT (×10^9^/L)	ρ= −0.08, *p* = 0.753	ρ = 0.34, *p* = 0.024	ρ = 0.32, *p* = 0.061
WBC (×10^9^/L)	ρ = 0.21, *p* = 0.387	ρ = 0.45, *p* = 0.003	ρ = 0.38, *p* = 0.025
ESR (mm/h)	ρ= −0.02, *p* = 0.926	ρ = 0.08, *p* = 0.821	ρ = 0.19, *p* = 0.511
hsCRP (mg/L)	ρ = 0.28, *p* = 0.334	ρ = 0.33, *p* = 0.054	ρ = 0.66, *p* < 0.001
*IL1B* (NRQ)	ρ = 0.33, *p* = 0.163	ρ= −0.09, *p* = 0.566	ρ = 0, *p* = 0.977
*TNFA* (NRQ)	ρ = 0.26, *p* = 0.286	ρ = 0.17, *p* = 0.271	ρ = 0.28, *p* = 0.102
*IL8* (NRQ)	ρ = 0.52, *p* = 0.023	ρ = 0.13, *p* = 0.384	ρ= −0.14, *p* = 0.429
*VEGFA* (NRQ)	ρ = 0.14, *p* = 0.567	ρ = 0.63, *p* < 0.0001	ρ = 0.48, *p* = 0.003

Data are presented as Spearman correlation coefficient (ρ). Hb, hemoglobin; PLT, platelet count; WBC, white blood cell count; ESR, erythrocyte sedimentation rate; hsCRP, high-sensitive C-reactive protein; *IL*, interleukin; *TNFA*, tumor necrosis factor; *VEGFA*, vascular endothelial growth factor; IBD, inflammatory bowel disease; CD, Crohn’s disease; UC, ulcerative colitis.

**Table 3:** Characteristics of study population—circulating Nampt/PBEF/visfatin.

Parameter	Non-IBD Controls		IBD		*p*
	Healthy Individuals	IBS	CD	UC	
N	40	20	113	127	-
Age, median (yrs.)	35 (25–44)	37.5 (23–50)	36 (28–47.25)	38 (28–52)	0.463 ^6^
Sex (F/M), n	20/20	12/8	58/55	55/72	0.419 ^7^
BMI, median (kg/m^2^)	25.3 (22.5–26.4) ^2^	26.2 (22.5–28.4) ^2^	22.2 (21.5–22.8) ^1,3,4^	23.9(22.9–24.8) ^2^	0.002 ^6^
Active disease, n (%)	-	-	74 (65.5)	55 (43.3)	<0.001 ^7^
Hb, median (g/dL)	na	13.3 (12.5–14.6) ^2^	12.3 (11.9–12.7) ^1^	12.7 (12.2–13.3)	0.048 ^6^
Iron, median (μM)	18.6 (16.4–21.6) ^2,3^	18.7 (12–24.8) ^2^	10.9 (8.7–11.8) ^1,3,4^	14.3 (12.3–16.3) ^2,4^	<0.001 ^6^
Tf, median (mg/dL)	276 (267–293) ^2^	284 (255–304) ^2^	236 (223–258) ^1,3,4^	263 (250–282) ^2^	0.009 ^6^
Alb, median (g/dL)	4.82 (4.7–4.9) ^2,3^	4.72 (4.6–4.9) ^2,3^	4.36 (4.2–4.4) ^1,3,4^	4.54 (4.4–4.6) ^1,2,4^	<0.001 ^6^
PLT, median (×10^9^/L)	na	261 (223–307)	343 (292–376)	293 (282–315)	0.094 ^6^
WBC, median (×10^9^/L)	na	6.55 (4.9–7.1)	7.0 (6.2–7.9)	6.87 (6.1–7.4)	0.773 ^6^
ESR, median (mm/h)	na	10 (5.5–24) ^2^	20 (18–26) ^1,3^	14 (11–20) ^2^	0.011 ^6^
hsCRP, median (mg/L)	2.21 (0.6–5.3) ^2,3^	1.47 (0–24) ^2,3^	16.6 (7.3–25.5) ^1,4^	5 (2–7) ^1,4^	<0.001 ^6^
IL-1β, median (pg/mL)	0.79 (0.39–2.04)	0.17 (0–1.28)	0.79 (0.47–1.25)	0.88 (0.70–1.43)	0.161 ^6^
IL-6, median (pg/mL)	0.68 (0.58–0.87) ^2,3^	1.11 (0.65–2.33) ^2^	2.74 (1.9–4) ^1,3,4^	1.98 (1.33–2.87) ^2,4^	<0.001 ^6^
TNFα, median (pg/mL)	1 (0.35–1.59)	0.62 (0–2)	0.77 (0.22–1)	0.49 (0.33–0.83)	0.780 ^6^
CS, n (%) ^5^	-	-	38 (53%)	34 (47%)	0.283 ^8^
AZA, n (%)	-	-	47 (42%)	30 (24%)	0.004 ^8^

If not otherwise stated, data are presented as means or medians with 95% confidence interval. BMI, body mass index; Hb, hemoglobin; Tf, transferrin; Alb, albumin; PLT, platelet count; WBC, white blood cell count; ESR, erythrocyte sedimentation rate; hsCRP, high-sensitive C-reactive protein; IL, interleukin; TNFα, tumor necrosis factor α; CS, corticosteroids; AZA, azathioprine; na, data non-available; ^1^, significantly different from IBS; ^2^, significantly different from CD; ^3^, significantly different from UC; ^4^, significantly different from controls; ^5^, calculated for patients with active disease; ^6^, Kruskall–Wallis H test; ^7^, Chi-squared test; ^8^, Fisher’s exact test.

**Table 4:** Characteristics of study population—leukocyte *Nampt/PBEF/visfatin*.

Parameter	IBS-controls	IBD		*p*
		CD	UC	
N	19	44	35	-
Age, mean (yrs.)	37.4 (30.7–45.6)	34.1 (31.2–37.3)	38.8 (34.5–43.6)	0.225 ^5^
Sex (F/M), n	7/12	18/26	15/20	0.912 ^6^
Active disease, n (%)	-	21 (48%)	20 (57%)	0.429 ^6^
Hb, mean (g/dL)	14.4 (13.7–15.1) ^1,2^	13 (12.3–13.6) ^3^	12.9 (12.1–13.8) ^3^	0.029 ^5^
PLT, median (×10^9^/L)	256 (212–294) ^1,2^	306 (244–337) ^3^	326 (280–367) ^3^	0.013 ^7^
WBC, median (×10^9^/L)	6.11 (5.53–6.97)	7.32 (5.38–8.95)	8.44 (7.29–9.5)	0.145 ^7^
ESR, median (mm/h)	7 (6–9) ^1,2^	23 (12–55) ^3^	21 (8–26) ^3^	<0.001 ^7^
hsCRP, mean (mg/L)	2 (0.8–5.2)	7.7 (3.9–15.2)	6.4 (2.9–14.3)	0.082 ^5^
CS, n (%) ^4^	-	7 (33%)	10 (50%)	0.285 ^8^
AZA, n (%)	-	18 (41%)	6 (18%)	0.047 ^8^

If not otherwise stated, data are presented as means or medians with 95% confidence interval. Hb, hemoglobin; PLT, platelet count; WBC, white blood cell count; ESR, erythrocyte sedimentation rate; hsCRP, high-sensitive C-reactive protein; IL, interleukin; TNF, tumor necrosis factor; CS, corticosteroids; AZA, azathioprine; ^1^, significantly different from CD; ^2^, significantly different from UC; ^3^, significantly different from controls; ^4^, calculated for patients with active disease; ^5^, one-way ANOVA; ^6^, Chi-squared test; ^7^, Kruskall–Wallis H test; ^8^, Fisher’s exact test.

**Table 5:** Primers’ sequences and efficiencies.

Symbol	Gene Name	Accession No.	Primer Sequence 5’→3’	Amp. Size (bp)	E (%) Leuko	E (%) Tissue
*SDHA*	Succinate dehydrogenase subunit A	NM_004168.2	F: agaggcacggaaggagtcac R: caccacatcttgtctcatcagtagg	267	94.8	-
*TBP*	TATA-box-binding protein	NM_003194.4	F: tataatcccaagcggtttgctg R: ctggctcataactactaaattgttg	283	109.7	-
*IL1B ^1^*	Interleukin (IL)-1β	NM_000576.2	F: ccacagaccttccaggagaatg R: gtgcagttcagtgatcgtacagg	131	100.1	94.7
*TNFA ^1^*	Tumor necrosis factor α	NM_000594.3	F: ctcttctgcctgctgcactttg R: atgggctacaggcttgtcactc	135	100.1	98.2
*IL8*	Interleukin 8	NM_000584.3	F: caacacagaaattattgtaaagc R: aagtgttgaagtagatttgc	191	-	96.7
*VEGFA ^1^*	Vascular endothelial growth factor A	NM_001025366.2	F: ttgccttgctgctctacctcca R: gatggcagtagctgcgctgata	126	94.5	96.1
*HIF1A*	Hypoxia-inducible factor 1α	NM_001530.3	F: ctgccaccactgatgaatta R: gtatgtgggtaggagatgga	90	-	104.7
*Nampt*	Nicotinamide phosphoribosyltransferase	NM_005746.2	F: cacaggcaccactaataatcagac R: ctccaccagaaccgaaggc	243	108.8	104
*RPS23 ^1^*	Ribosomal protein S23	NM_001025.4	F: aggaagtgtgtaagggtccagc R: caccaacagcatgacctttgcg	142	-	102.9
*PPIA ^1^*	Peptidylprolyl isomerase A	NM_021130.3	F: ggcaaatgctggacccaacaca R: tgctggtcttgccattcctgga	161	-	99.7
*RPLP0 ^1^*	Ribosomal protein, large, P0	NM_001002.3	F: tcacaacaagcataccaagaagc R: gtatccgatgtccacaatgtcaag	263		102.1
*FGF2*	Basic fibroblast growth factor	NM_002006.5	F: tctatcaaaggagtgtgtgctaac R: tgcccagttcgtttcagtgc	179	-	100.7

Amp., amplicon; E, efficiency; leuko, leukocyte expression; ^1^, primer sequences were as proposed by Origene (www.origene.com). Remaining primers were designed using Beacon Designer Probe/Primer Design Software (BioRad), validated in silico by Blast analysis, and their specificity tested by means of melting curve analysis and an electrophoresis in a high-resolution agarose (SeaKem LE agarose, Lonza, Switzerland) in TBE with SYBR Green (Lonza) detection. Efficiencies were calculated on pooled cDNA, separately for expression analysis in whole blood and bowel tissue experiments. Forward and reverse primer sequences are denoted by “F” and “R”, respectively.

## Supplementary Materials

The supplementary materials are available online at http://www.mdpi.com/1422-0067/20/1/166/s1.

## Abbreviations

Nampt	Nicotinamide phosphoribosyltransferase
PBEF	pre-B-cell colony enhancing factor 1
IBD	inflammatory bowel disease
UC	ulcerative colitis
CD	Crohn’s disease
IBS	irritable bowel syndrome
CDAI	Crohn’s Disease Activity Index
RI	Rachmilewitz Index
CS	corticosteroids
AZA	azathioprine
hsCRP	high-sensitive C-reactive protein
CRC	colorectal adenocarcinoma
ROC	Receiver operating characteristic curve analysis
AUC	area under ROC curve
MH	mucosal healing
