# Supplementary material for: Oversecretion and Overexpression of Nicotinamide Phosphoribosyltransferase/Pre-B Colony-Enhancing Factor/Visfatin in Inflammatory Bowel Disease Reflects the Disease Activity, Severity of Inflammatory Response and Hypoxia
Source: Int J Mol Sci. 2019 Jan 4;20(1):166. doi: 10.3390/ijms20010166 (PMC6337260; doi:10.3390/ijms20010166)

Supplementary Figures

Fig. 1 Correlation betwen serum Nampt (S-Nampt) and Rachmilewitz index (RI)(panel A) and between S-Nampt and endoscopic score (panel B) (Spearman correlation test)

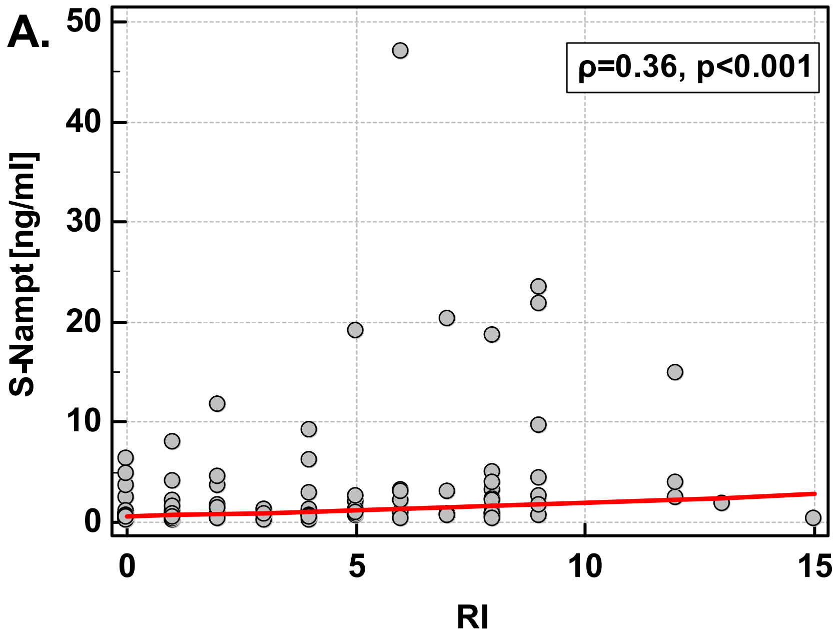

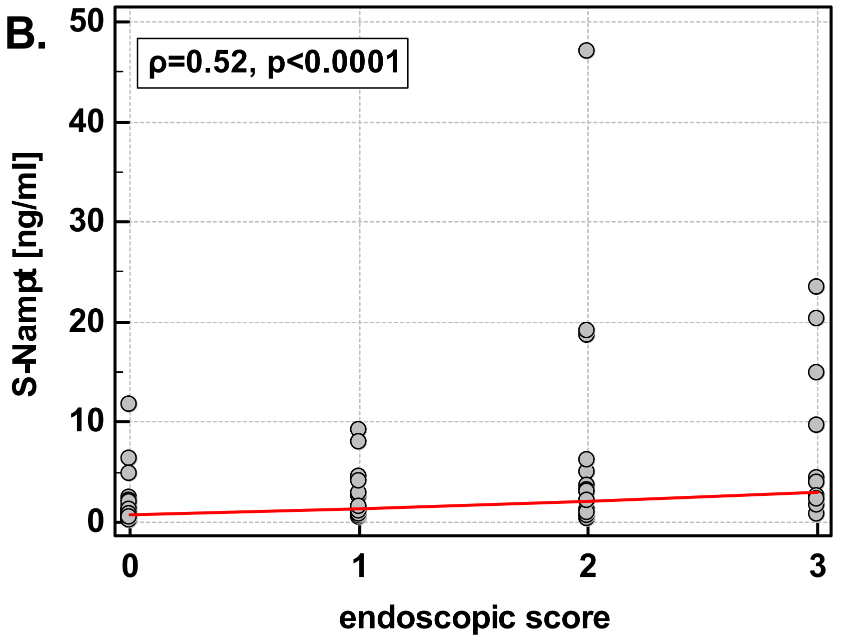

Fig 2. Correlation of serum Nampt (S-Nampt) with circulating cytokines in patients with active ulcerative colitis (Pearson correlation test)

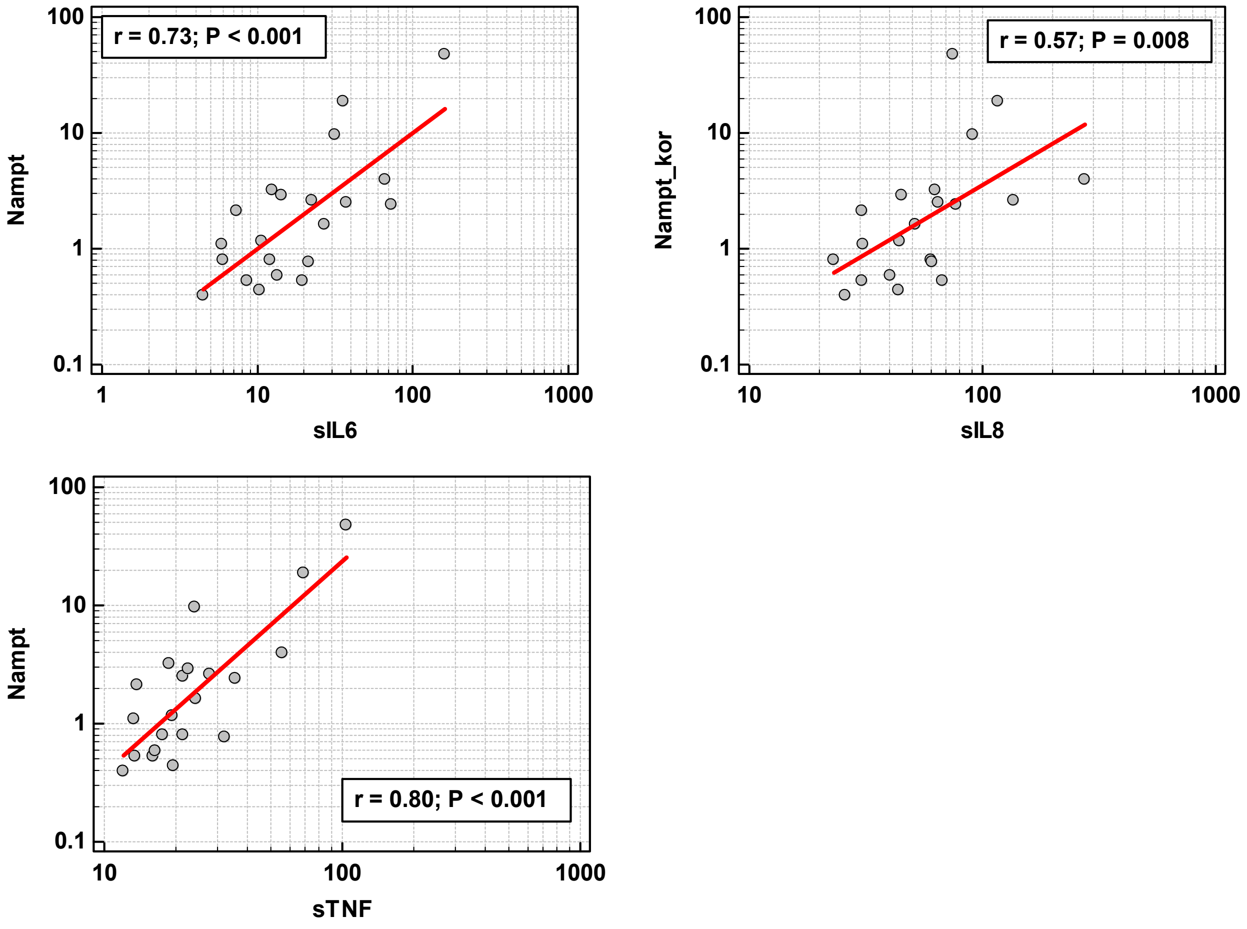

Fig 3. Correlation of leukocyte Nampt (L-Nampt) with circulating cytokines in patients with IBD
(Pearson correlation test)

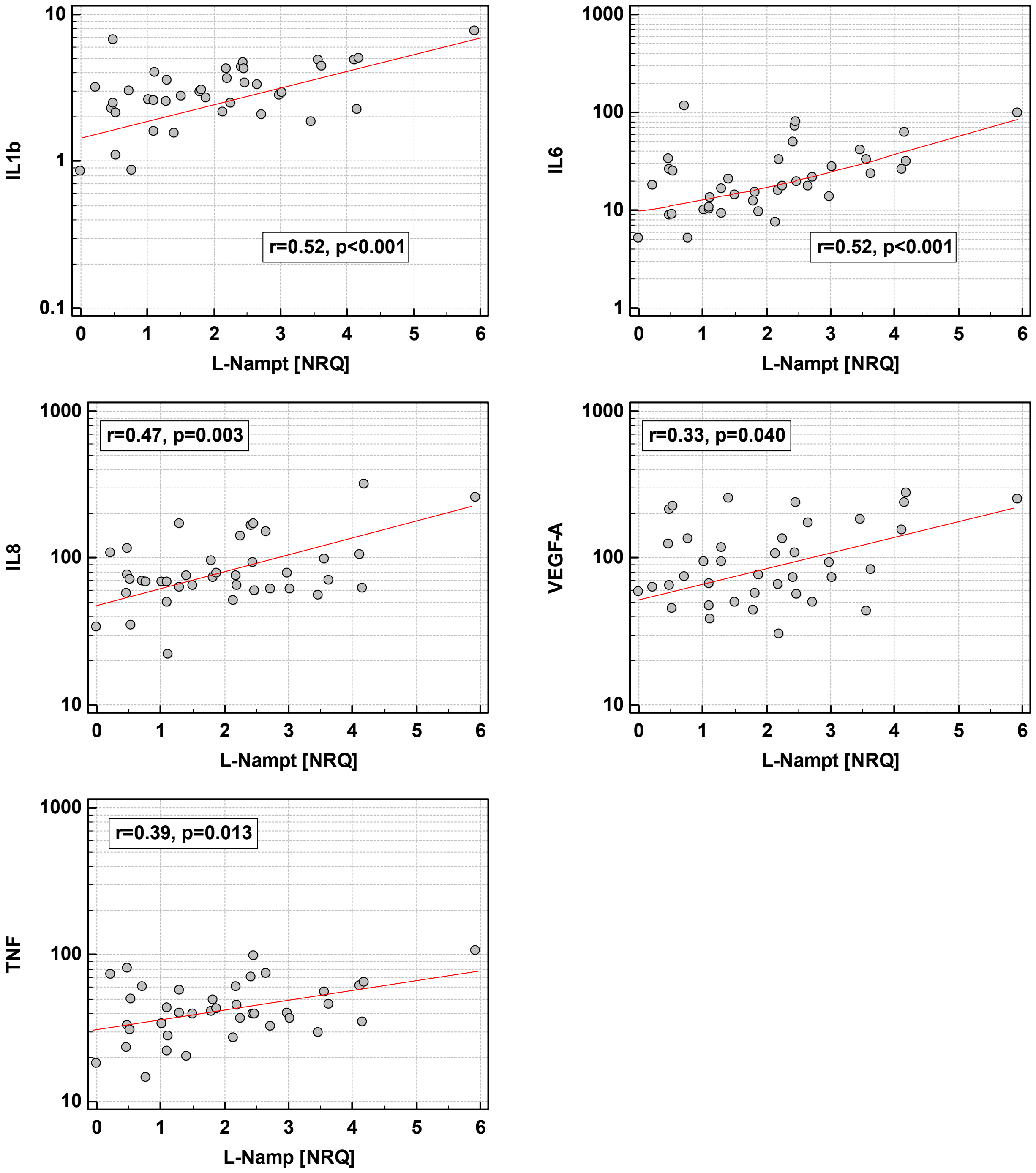

Fig 4. Correlation of leukocyte Nampt (L-Nampt) with circulating cytokines in patients with active IBD (Pearson correlation test)

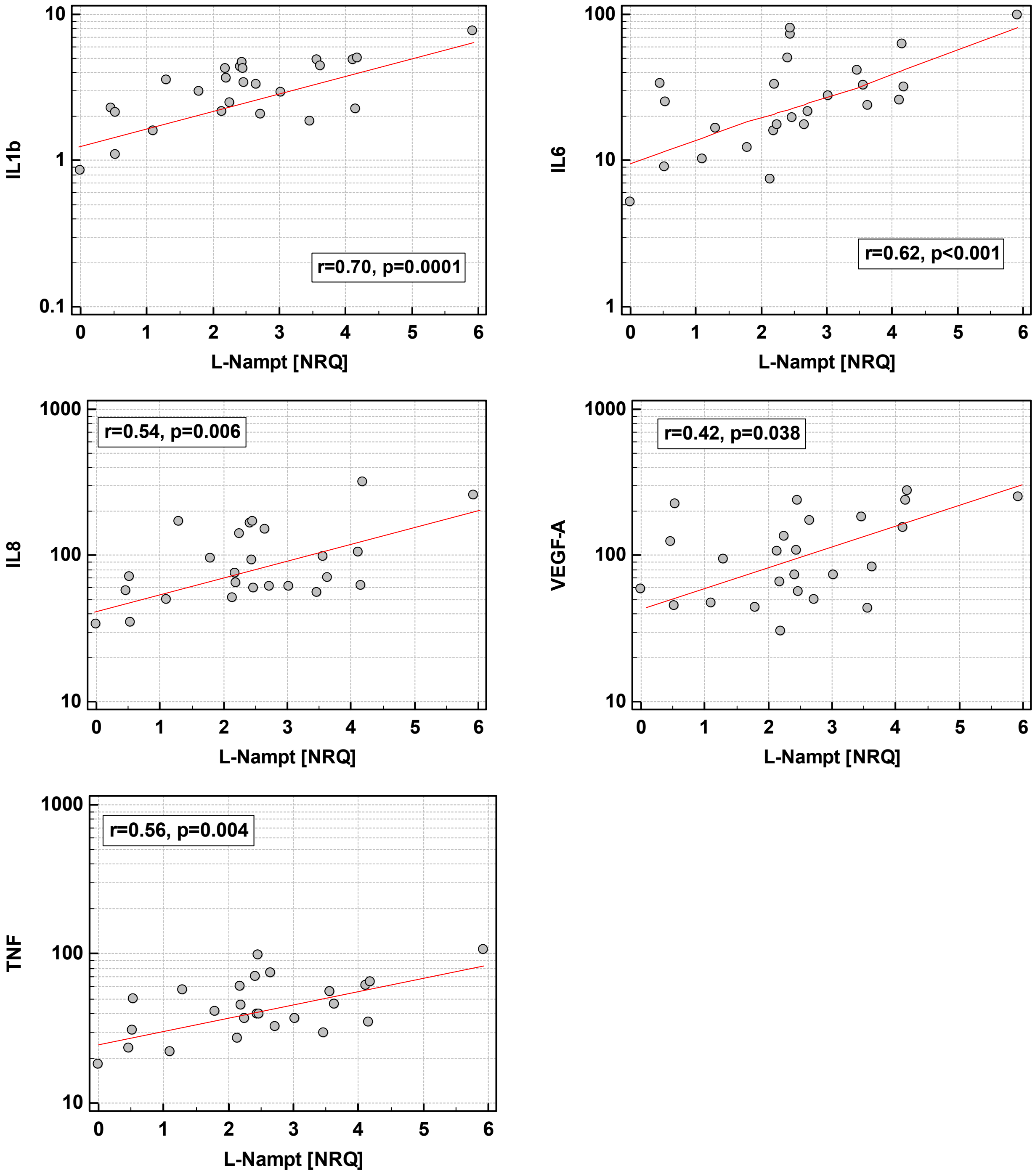

Fig 5. Correlation of leukocyte Nampt (L-Nampt) with circulating Nampt (S-Nampt) (Pearson correlation test)

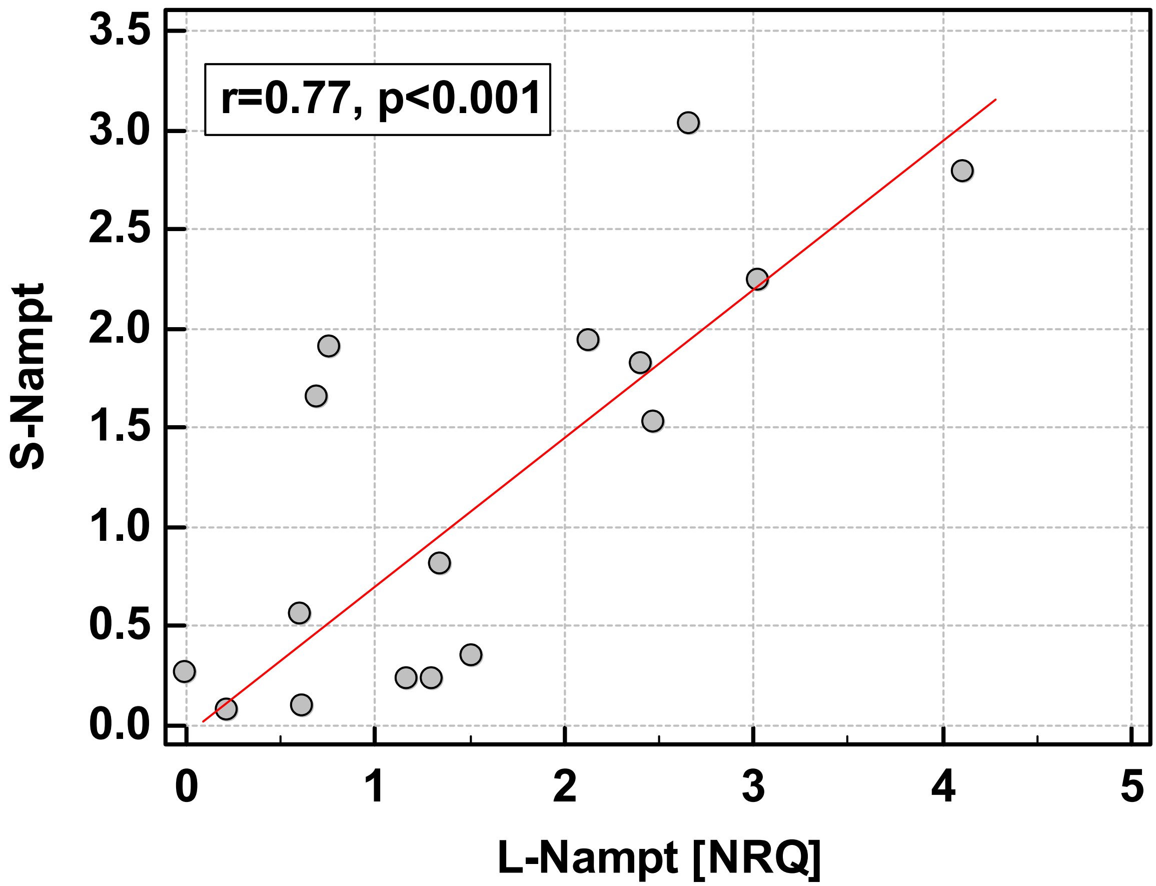

Fig. 6 Correlation of bowel Nampt (b-Nampt) with bowel expression of selected cytokines and transcription factor HIF1a in inflamed small bowel

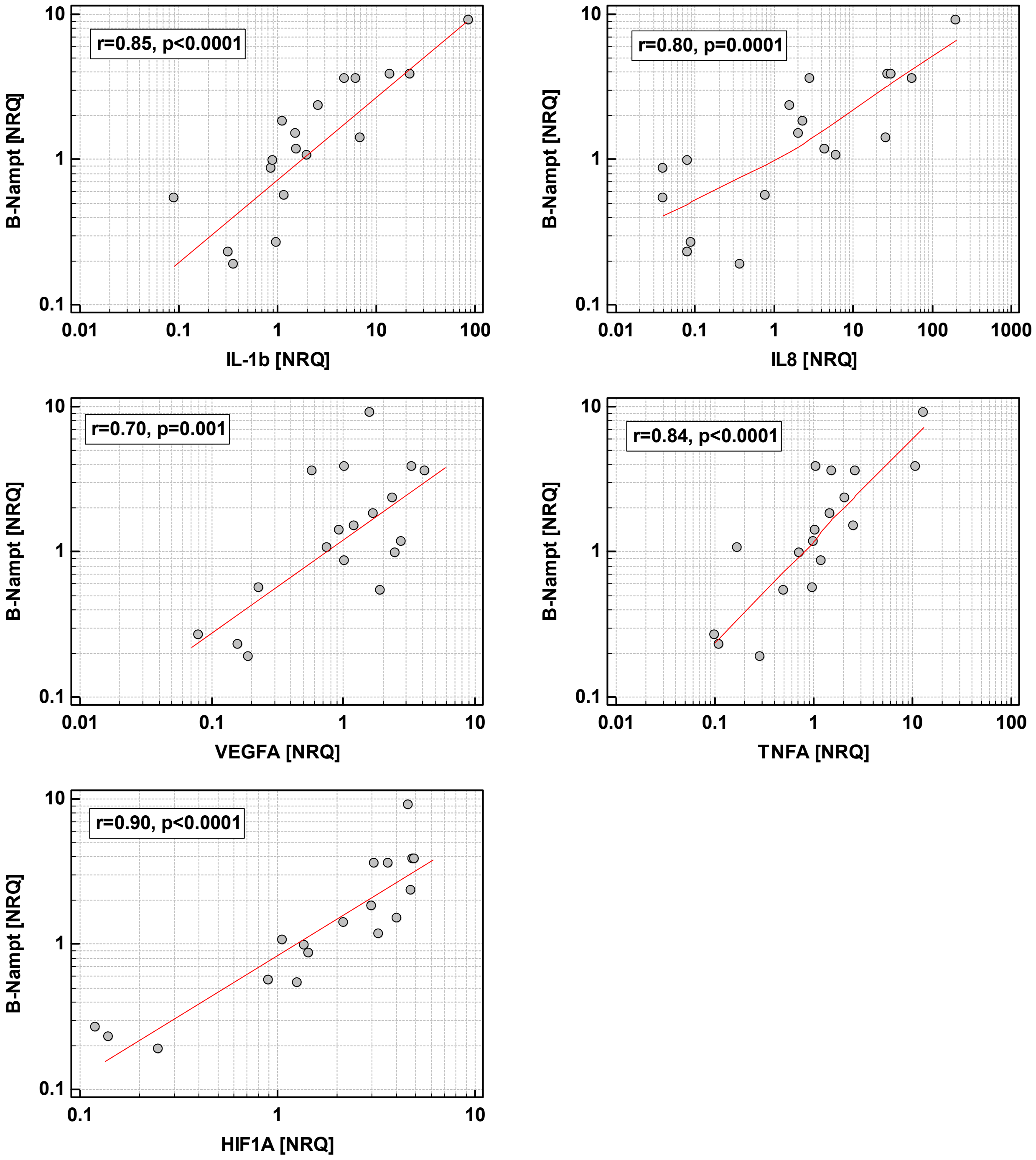

Fig. 7 Correlation of bowel Nampt (b-Nampt) with bowel expression of selected cytokines and transcription factor HIF1a in quiescent small bowel

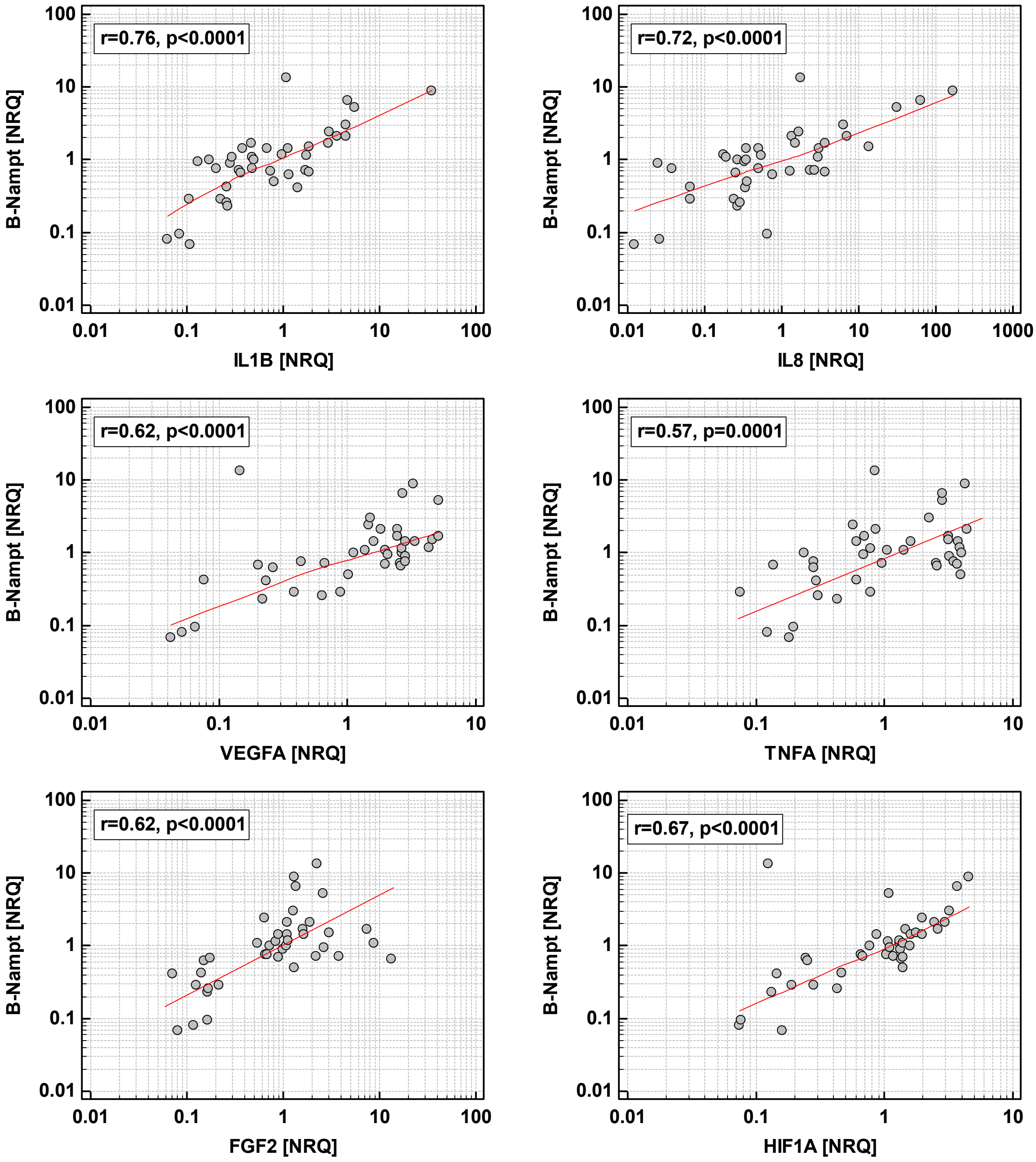

Fig 8 Correlation of bowel Nampt (b-Nampt) with bowel expression of selected cytokines and transcription factor HIF1a in inflamed large bowel

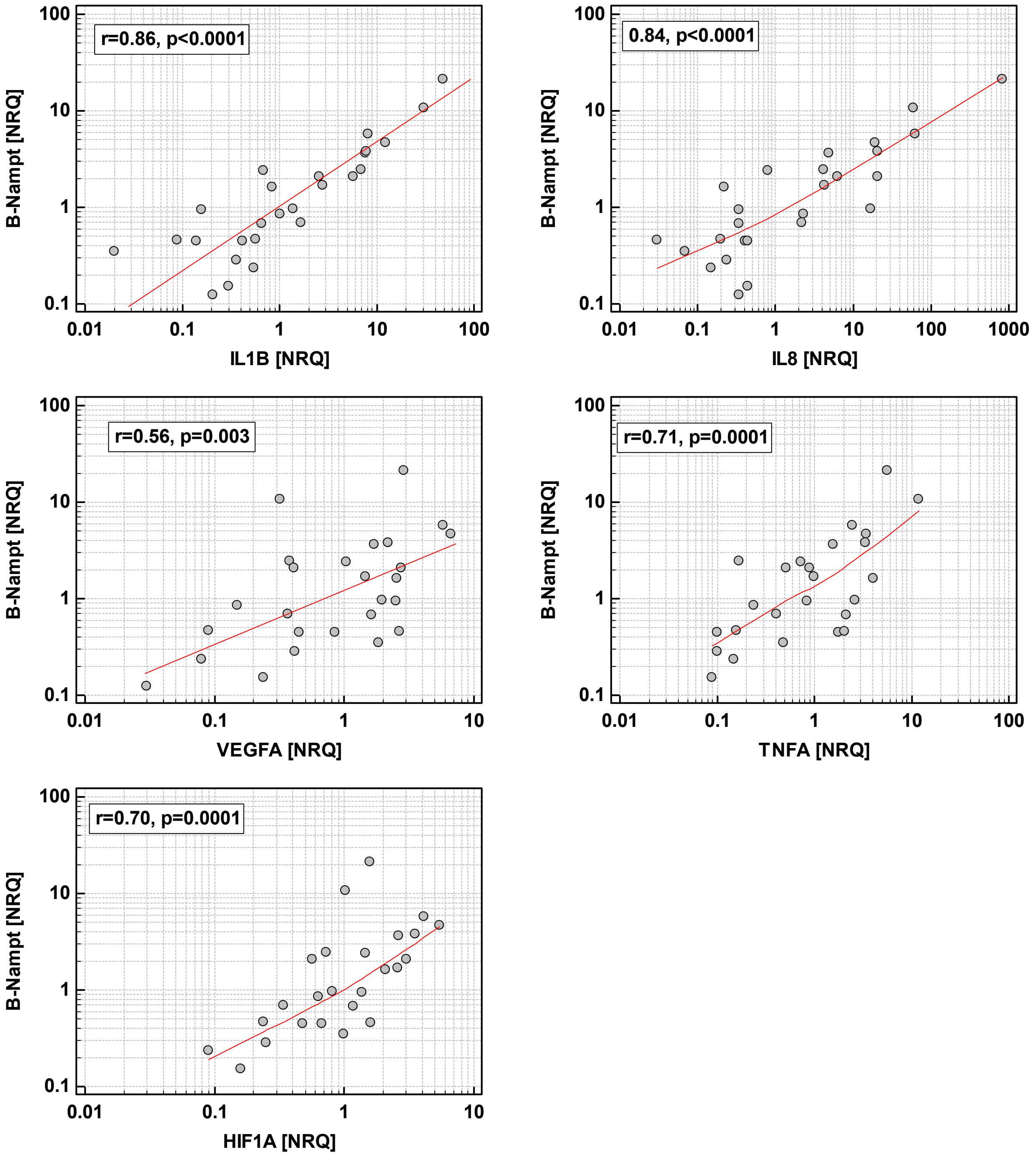

Fig 9 Correlation of bowel Nampt (b-Nampt) with bowel expression of selected cytokines and transcription factor HIF1a in quiescent large bowel

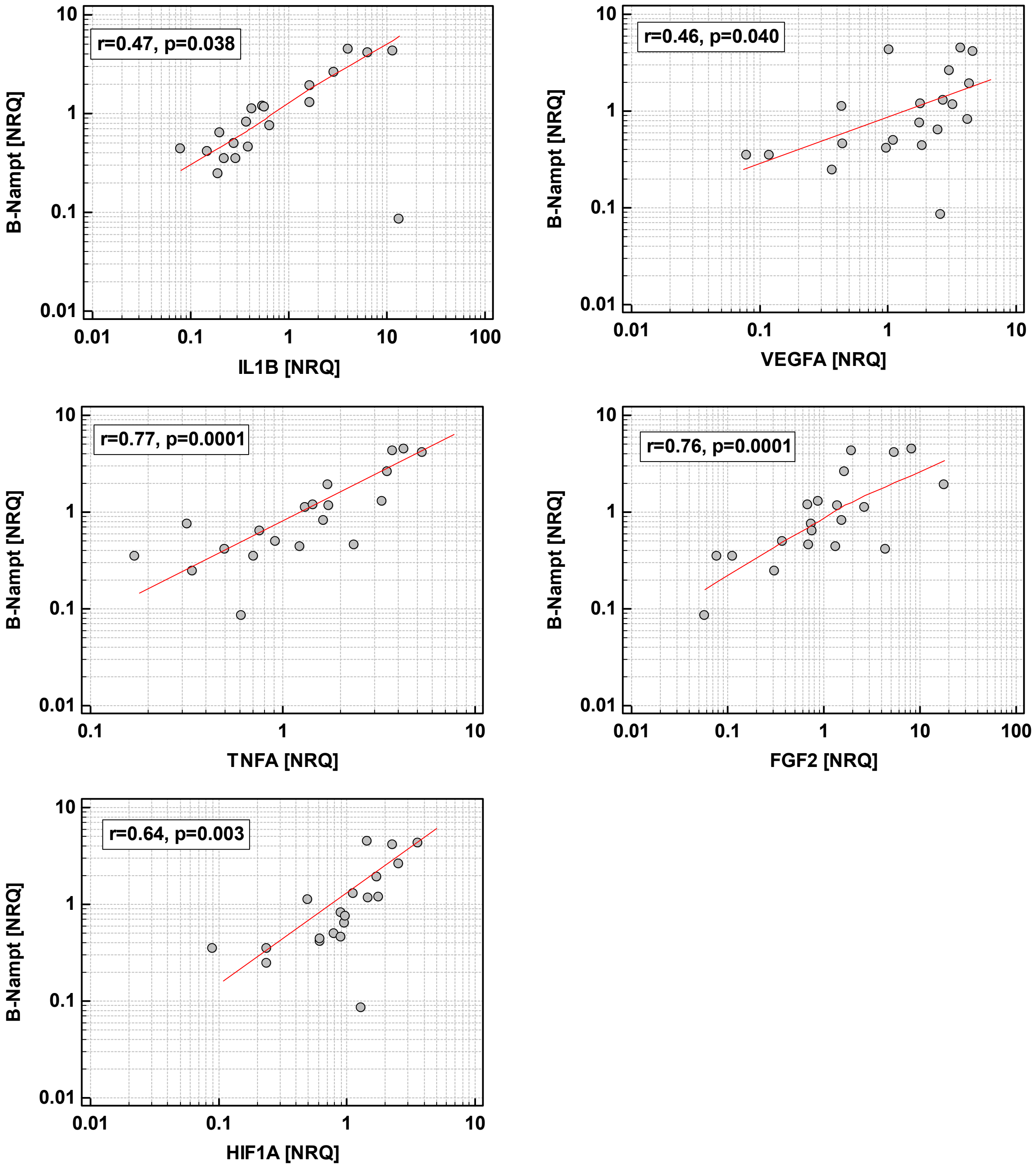

Supplement: Supplementary file 1 [file ijms-20-00166-s001.pdf]
